# Eco-friendly synthesis of nZVI for water remediation: latest developments and environmental applications

**DOI:** 10.1039/d6ra02489k

**Published:** 2026-05-21

**Authors:** Basem E. Keshta, Tesnim Dhiss, Jing Yu, Qiaoping Kong, Huma Javeria, Yasmeen G. Abou El-Reash, Heba G. El-Attar, Hany Koheil, Eida S. Al-Farraj, Mohamed N. Goda, Antonio Cid-Samamed

**Affiliations:** a Chemistry Department, Faculty of Science, Tanta University Tanta 31512 Egypt basem.keshta@science.tanta.edu.eg YGElReash@imamu.edu.sa; b University of Gabès, National School of Engineers of Gabès, Laboratory of Research: Processes, Energy, Environment & Electrical Systems PEESE (LR18ES34) Rue Omar Ibn Alkhattab 6029 Gabès Tunisia; c School of Environmental and Municipal Engineering, Qingdao University of Technology Qingdao 266520 PR China; d Department of Toxicology Pusat Kanser Tun Abdullah Ahmad Badawi (PKTAAB) Universiti Sains Malaysia (USM) 13200 Bertam Kepala Batas Pulau Pinang Malaysia; e Department of Chemistry, College of Science, Imam Mohammad Ibn Saud Islamic University (IMSIU) P.O. Box, 90950 Riyadh 11623 Saudi Arabia; f Physics and Engineering Mathematics Department, Faculty of Engineering, Kafr Elsheikh University Kafr Elsheikh 33516 Egypt; g Physical Chemistry Department, Faculty of Sciences, University of Vigo E-32004 Ourense Spain

## Abstract

This review discusses recent advances in the green synthesis of nanoscale zero-valent iron (nZVI), with emphasis on plant-derived materials as sustainable reducing and stabilizing agents. A wide range of plant extracts obtained from leaves, seeds, peels, and other biomass sources have been employed to generate nZVI through phytochemical-mediated reduction, offering an eco-friendly alternative to conventional chemical methods. Emerging research demonstrates a clear shift toward bio-inspired and circular synthesis strategies, where polyphenol- and alkaloid-rich extracts enhance particle stability, control size distribution (typically 10–100 nm), and improve reactivity. Moreover, these research strategies have pointed out a notable increase in the reusability of nZVI and have also been directed toward integrating nZVI compounds with natural supports, such as clays, biochar, and agricultural residues, to limit aggregation, increase reusability, and improve performance in complex wastewater matrices. Green-synthesized nZVI has shown broad potential in sustainable remediation, with particle sizes generally ranging from 10 to 80 nm and removal efficiencies of approximately 90% for dyes, heavy metals, and antibiotics under optimized conditions. Supported nZVI composites further demonstrate strong environmental robustness, exhibiting up to 95% reusability over multiple cycles. The novelty of this review lies in its integrated analysis connecting synthesis routes, physicochemical properties, and environmental performance of plant-mediated nZVI, addressing gaps in earlier literature that largely overlooked these correlations. Finally, the review critically evaluates the scalability, stability, and ecological impacts of green-synthesized nZVI and outlines future research needs for improving reproducibility and advancing practical applications in real-world water treatment systems.

## Abstract 2

This review discusses recent advances in the green synthesis of nanoscale zero-valent iron (nZVI), with emphasis on plant-derived materials as sustainable reducing and stabilizing agents.

## Graphical abstract

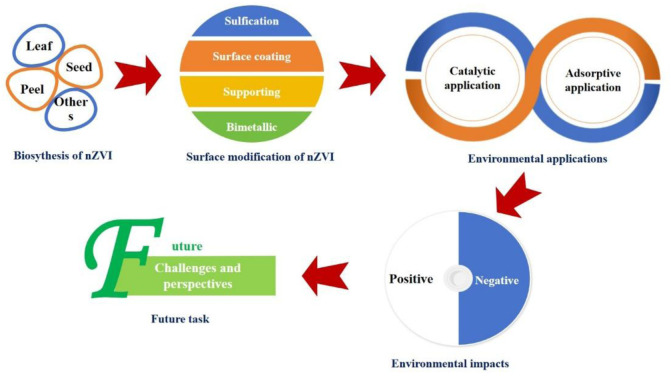

## 1. Introduction

Nanotechnology, which involves manipulating matter at the nanoscale (1–100 nm), has introduced transformative innovations across various fields due to the unique physicochemical properties of nanomaterials.^1–3^ Among these, zero-valent iron (nZVI) has emerged as a viable candidate for environmental remediation owing to its high surface reactivity, strong electron-donating capability, and ability to degrade a broad spectrum.^4–6^

Nanoparticle synthesis typically follows either top-down or bottom-up approaches.^7^ While top-down methods involve the physical fragmentation of bulk materials, bottom-up techniques such as chemical reduction and sol–gel processes build nanostructures from molecular or atomic precursors. However, conventional bottom-up synthesis often relies on hazardous chemical reducing agents (*e.g.*, sodium borohydride, hydrazine), raising serious concerns regarding environmental toxicity and safety.^8^

As a sustainable alternative, green synthesis techniques have gained increasing attention.^9–13^ These methods utilize biological systems or natural products as reducing and stabilizing agents, offering environmentally benign, cost-effective, and scalable pathways for nanoparticle production.^14,15^ A diverse range of biological resources including bacteria,^16^ algae,^17^ biomolecules,^18^ agricultural wastes,^19^ and particularly plant extracts have shown potential in nanoparticle biosynthesis.^20^

**Fig. 1:**
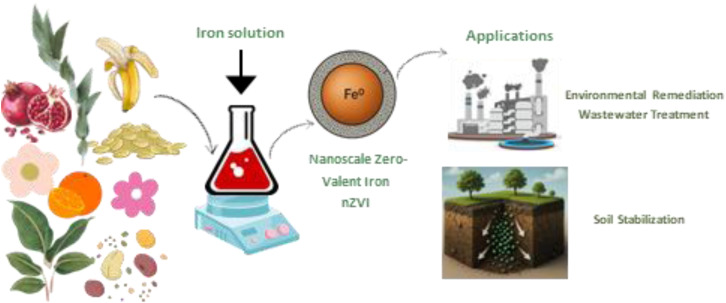
Green synthesis of nZVI from plant extracts for environmental applications.

Plants are especially attractive for nZVI synthesis due to their rich content of phytochemicals (polyphenols, flavonoids, alkaloids, *etc.*) that naturally reduce metal ions and cap the resulting nanoparticles.^21,22^ Several studies have reported the successful use of plant parts such as leaves, seeds, peels in the synthesis of nZVI, producing nanoparticles with tunable morphologies and enhanced reactivity.^23^ A visual overview of this green synthesis pathway and its environmental applications is provided on Fig. 1. Owing to its strong reducing power and large surface area, nZVI has been extensively applied for the removal of environmental contaminants.

Water pollution remains one of the most pressing global environmental challenges, driven by the continuous release of industrial effluents, agricultural runoff, and emerging contaminants into aquatic systems.^24–27^ To address these issues, numerous treatment materials have been investigated, including metal–organic frameworks (MOFs)^28^,^29^, clay minerals,^30^ TiO_2_ photocatalysts^31^,^32^, magnetic adsorbents,^33^,^26^ and various engineered nanomaterials,^34,35^. Among these, nZVI has emerged as a particularly effective option due to its strong reductive capacity, broad contaminant applicability, and relatively low cost.^36^ In addition to its reducing chemical inputs and promoting environmentally benign production.^37^ These advantages position green-synthesized nZVI as a promising and sustainable material for advanced water treatment and pollution control.^25^

Despite its advantages, nZVI faces several challenges that limit its effectiveness in environmental applications, such as aggregation, passivation, limited mobility, and reduced electron transfer efficiency.^38,39^ To overcome these limitations, researchers have investigated strategies such as supporting nZVI on different substrates, including clay,^40^ carbon/activated carbon,^41^ doping of nZVI with other metals like copper,^42^ and developing bimetallic composites^43^ and biochar- Cu/nZVI.^44^

This review provides a concise, critical assessment of recent advances in the green synthesis of nZVI, with a focus on planted routes as sustainable alternatives to conventional chemical methods. It elucidates how phytochemical composition controls nanoparticle size, morphology, stability, and mechanistic pathways, and links these features to environmental performance, particularly in wastewater remediation. By correlating plant extract chemistry with nZVI structure, texture, and remediation efficiency, and by comparing green and conventional synthesis under realistic operational constraints, the review offers a mechanistic and application-oriented framework to guide future scalable and environmentally responsible nZVI production and use.

## 2. Scope and methodology within the environmental context of nZVI

nZVI has emerged as one of the most widely studied engineered nanomaterials for environmental remediation owing to its strong reducing power, large specific surface area, and high surface reactivity.^45–48^ The Fe^0^/Fe^2+^ redox couple (*E*° ≈ −0.44 V *vs.* SHE) and the abundance of reactive surface sites enable rapid electron transfer to reducible contaminants, including chlorinated solvents, hexavalent chromium, nitroaromatic compounds, dyes, and various emerging organic pollutants.^49–52^ These redox and surface properties underpin both direct electron-transfer reactions and indirect oxidative pathways mediated by iron corrosion products such as Fe^2+^ and Fe^3+^ species, which participate in Fenton-like reactions to generate reactive oxygen species and broaden nZVI's remediation versatility.^53^

Mechanistically, nZVI operates through several complementary routes: (i) direct reduction of target compounds *via* electron transfer from Fe^0^; (ii) adsorption and co-precipitation of metal cations with iron oxides and hydroxides formed during corrosion; and (iii) indirect oxidation in advanced oxidation processes (AOPs), where Fe^2+^ catalyses H_2_O_2_ activation to hydroxyl radicals.^54^ This combination of pathways enables nZVI to address a wide range of inorganic and organic contaminants, supporting its use as both a primary reductant and as a catalyst or promoter in hybrid chemical–biological systems.^55,56^

A further advantage of nZVI lies in its tunability. Surface modification strategies such as sulfidation, carbon or polymer coating, and doping with other metals can mitigate aggregation and passivation while enhancing stability, selectivity, and reactivity.^57,58^ Similarly, immobilization on natural supports, such as biochar, clays, or activated carbon, improves dispersibility and transport in porous media, allowing the material to be recovered and reused after treatment.^59–62^ These engineered composites have shown higher removal efficiency and longer reactive lifetimes than bare nZVI, illustrating the importance of support and surface design.

In addition, nZVI can synergize with biological processes. By modifying redox conditions, releasing Fe^2+^ ions, or enhancing contaminant bioavailability, nZVI can stimulate microbial activity and promote reductive biotransformation. Several studies report enhanced bioremediation efficiency when nZVI is used in conjunction with microbial consortia or organic amendments, particularly in soils and sediments where both abiotic and biotic reduction contribute to pollutant removal.^63,64^

Finally, for practical field deployment, it is essential to consider mobility, ageing, and secondary mineral formation. nZVI's reactivity and lifetime can be influenced by pH, ionic strength, and the presence of dissolved oxygen or natural organic matter. Green approaches, especially plant-mediated routes, aim to address these challenges by providing surface-stabilized particles with reduced toxicity, lower cost, and improved environmental compatibility.^65–67^ In summary, the combination of (i) intrinsic reductive reactivity, (ii) mechanistic versatility, (iii) amenability to surface engineering, and (iv) compatibility with biological processes establishes nZVI as a strategically important material for both laboratory and field-scale remediation efforts.

Also, this review will cover three interconnected dimensions: (i) the diversity of plant-derived extracts used as natural reducing and stabilizing agents; (ii) the relationships between phytochemical composition, resulting structural and textural features of nZVI, and their mechanistic roles in pollutant degradation; and (iii) emerging trends in surface modification, composite formation, and hybrid bio–nano systems that enhance stability, reactivity, and reusability under real wastewater conditions. Additionally, the review assesses the sustainability impacts of green nZVI using circular economy principles and relevant Sustainable Development Goals (SDGs), positioning this technology within a broader environmental and socio-economic context.

Only peer-reviewed studies reporting experimental synthesis, physicochemical characterization, or pollutant removal performance of biologically derived nZVI were included. Approximately over 200 relevant publications were systematically analyzed to compare synthesis routes, nanoparticle characteristics, mechanistic insights, and environmental applications. This methodological approach ensures a robust and integrative evaluation of current knowledge, enabling the identification of research gaps, technological bottlenecks, and emerging opportunities for the sustainable development of green nZVI.

nZVI enhances bioremediation by altering redox conditions and releasing Fe^2+^, which serves as an electron donor and enzymatic cofactor for microbial reductive processes. Through the Fe^0^/Fe^2+^/Fe^3+^ redox cycle, nZVI drives sequential electron transfer, enabling direct contaminant reduction and Fenton-like ROS generation. Simultaneously, partial abiotic reduction and reactive Fe(ii)/Fe(iii) (hydr)oxide interfaces convert pollutants into more bioavailable intermediates, sustaining coupled abiotic–biotic degradation.

Although polyphenols and flavonoids are frequently cited as the primary drivers of green nZVI formation, their role extends beyond simple reduction. High-molecular-weight tannins and proteins tend to produce thicker organic shells that enhance colloidal stability but reduce electron-transfer efficiency, whereas low-molecular-weight phenolics favor faster nucleation and higher Fe^0^ exposure. This explains why extracts such as green tea and eucalyptus consistently yield smaller, more reactive particles, while seed- or peel-based extracts often produce larger but more stable nZVI. Therefore, extracting molecular weight distribution, rather than total phenolic content alone, governs the balance between reactivity and stability.

## 3. Biosynthesis of nZVI using various plant extracts

The biosynthesis of nZVI using plant extracts has emerged as a promising green alternative to conventional chemical synthesis methods, which has gained growing interest at home and abroad.^68^ Unlike traditional synthesis routes that rely on hazardous chemical reducing agents such as sodium borohydride or hydrazine, plant-mediated synthesis utilizes naturally occurring phytochemicals found in various plant tissues. These include polyphenols, amino acids, alkaloids, terpenoids, flavonoids, phenolic compounds, and sugars such as glucose, which function as both reducing and stabilizing agents during formation of nZVI.^69–71^

Although the rate of nZVI formation through plant-mediated synthesis may be slower and may yield particles with less uniform dispersion compared to conventional chemical methods, the environmental advantages of this green approach are significant. Notably, it minimizes the production of toxic byproducts and avoids the use of energy–intensive processes. Additionally, the presence of bio-organic compounds from plant extracts on the nanoparticle surface will affect the stability, dispersibility, and reactivity of nZVI in environmental applications. For example, it is found that nZVI synthesized from plant extracts with high polyphenol content can reduce the size of aggregates and improve the fluidity of particles in applications such as groundwater remediation.^72^ Thus, the biosynthesis of nZVI using plant extracts may represent a viable and sustainable route, particularly relevant to environmental remediation technologies where green chemistry principles are increasingly emphasized.

Moreover, the textural and morphological characteristics of green-synthesised nZVI are strongly dependent on the biochemical composition of the plant extract and synthesis parameters. Reported BET surface areas typically range between 25 and 65 m^2^ g^−1^, while particle sizes fall within 10–80 nm, both of which significantly influence reactivity and pollutant removal performance.^15,73^ For example, polyphenol-rich extracts from green tea, eucalyptus, or pomegranate have yielded smaller, well-dispersed nanoparticles with higher surface areas, leading to enhanced degradation efficiencies of > 90% for dyes and antibiotics.^37,74,75^ Conversely, extracts with lower reduced capacity often produce aggregated particles with limited active sites and lower reactivity. Supporting nZVI on porous matrices such as biochar, clay, or activated carbon further improves textural properties, achieving surface areas up to 120 m^2^ g^−1^ and maintaining over 95% reusability after five cycles, demonstrating the critical link between structure and catalytic stability.^76^ Therefore, optimizing synthesis parameters to tailor surface area, porosity, and particle dispersion is essential to improving the performance and durability of green nZVI for real-world environmental remediation.

A comparative summary of the principal green synthesis strategies for nZVI is provided in Table 1. The table compiles information already discussed in the text, highlighting the typical precursors and reducing agents, relative scalability, and main advantages and limitations of each route. This summary facilitates direct comparison of the diverse approaches reported in the literature and underscores their respective potential for sustainable implementation. In conclusion, while nZVI-based technologies offer significant potential for environmental remediation, addressing these challenges through interdisciplinary research, technological innovation, and regulatory development will be essential for their sustainable and responsible implementation.

**Table 1:** Key green route for synthesizing nZVI and their corresponding precursors

Green synthesis route	Typical precursor/reductant	Scalability	Advantages	Limitations	Ref.
Plant extract-mediated	Polyphenols, flavonoids, tannins	Moderate to high	Renewable, cost-effective, tunable particle size	Variability of extract composition	23, 60, 77 and 78
Microbial-assisted	Bacterial or fungal metabolites	Low	Biocompatible, mild conditions	Slow kinetics, culture maintenance	
Polysaccharide-assisted	Starch, cellulose, chitosan	Moderate	Natural stabilisation, good dispersion	Limited scalability	17
Waste biomass-derived	Agricultural or food residues	High	Valorization of residues, circular economy	Impurities, standardisation	58 and 79

While green-synthesized nZVI is often promoted as a sustainable alternative to borohydride-reduced nZVI, a critical comparison reveals clear performance trade-offs. Chemically synthesized nZVI typically exhibits smaller primary particle sizes (5–30 nm), higher Fe^0^ content, and faster initial reduction kinetics, whereas green-synthesized nZVI commonly shows broader size distributions (20–100 nm) and partial surface oxidation due to phytochemical capping layers. Quantitative comparisons across studies indicate that, under identical laboratory conditions, borohydride-reduced nZVI often achieves 10–30% higher apparent removal rates for rapidly reducible contaminants such as Cr(vi) and chlorinated solvents. However, green-synthesized nZVI demonstrates superior colloidal stability, lower aggregation rates, and enhanced reusability, particularly when supported on biochar or clay matrices. These differences highlight that green synthesis does not universally outperform chemical routes, but instead shifts the performance balance toward stability, safety, and sustainability at the expense of peak reactivity.

As discussed in Section 2, nZVI mediates contaminant removal through several complementary mechanisms, including direct reductive degradation *via* electron transfer from the Fe^0^ core, adsorption and co-precipitation on iron oxyhydroxide corrosion products, and indirect oxidative degradation through Fenton-like generation of reactive oxygen species (·OH, O_2_˙^−^). These fundamental pathways operate regardless of the synthesis route; however, the choice of plant extract and synthesis conditions can significantly modulate their relative contributions by influencing particle size, surface chemistry, Fe^0^ content, and organic capping layer thickness. The following subsections examine how specific plant-derived extracts have been employed in nZVI synthesis and how the resulting physicochemical properties govern remediation performance.

### 3.1 Systematic comparison of green *vs.* chemically synthesized nZVI

A meaningful evaluation of green nZVI requires direct comparison with its chemically synthesized counterpart across multiple performance dimensions. In terms of particle characteristics, borohydride-reduced nZVI consistently produces smaller primary particles (5–30 nm) with higher specific surface areas (typically 25–50 m^2^ g^−1^) and greater Fe^0^ content (60–90 wt%), owing to the strong and rapid reduction by NaBH_4_.^80,81^ Green-synthesized nZVI, by contrast, exhibits broader size distributions (20–100 nm), lower Fe^0^ content (30–70 wt%), and partial surface oxidation due to residual phytochemical capping layers.^82^ These structural differences translate directly into kinetic behaviour: chemically synthesized nZVI typically achieves 10–30% faster initial removal rates for rapidly reducible contaminants such as Cr(vi) and trichloroethylene under controlled laboratory conditions.^83–85^ Green-synthesized analogues, however, deliver only 70–90% of the initial reductive rate but exhibit superior long-term stability and reusability owing to the phytochemical capping that mitigates surface passivation.^84^

However, green-synthesized nZVI demonstrates clear advantages in colloidal stability and longevity. The organic capping layer, often composed of polyphenols and flavonoids, provides steric and electrostatic stabilization that significantly reduces aggregation rates compared to bare chemical nZVI, which aggregates rapidly in the absence of polymeric stabilizers [102–104]. This enhanced dispersibility translates to superior reusability: supported green nZVI composites commonly retain 70–95% removal efficiency over 3–5 cycles, whereas unsupported chemical nZVI typically loses >50% reactivity after 2–3 cycles due to rapid oxidation and passivation.^86,87^

Ageing behaviour represents another key differentiator. Chemical nZVI undergoes rapid surface passivation through oxidation to magnetite (Fe_3_O_4_) and goethite (α-FeOOH) within days to weeks under oxic conditions, substantially diminishing its reactive lifetime. Green nZVI ages more slowly, as the organic shell partially shields the Fe^0^ core from water and dissolved oxygen, extending functional longevity.^78,88^ Sulfidated variants of both types (S-nZVI) show markedly improved ageing resistance regardless of synthesis route.

Perhaps the most critical and least studied dimension is performance in realistic water matrices. Both synthesis types suffer significant performance reductions (20–50%) when transitioning from deionized water to real wastewater or groundwater, due to competition from co-existing ions (HCO_3_^−^, SO_4_^2−^, Ca^2+^), natural organic matter fouling, and pH variability. However, supported green nZVI composites (*e.g.*, biochar- or clay-supported) show comparatively better resistance to matrix effects, likely due to the additional adsorptive capacity and buffering provided by the support material.^89,90^ Systematic head-to-head studies under identical real-water conditions remain scarce and represent a critical research need.

In summary, the two synthesis approaches do not represent a simple hierarchy but rather a performance trade-off: chemical nZVI excels in peak reactivity and initial kinetics, while green nZVI offers superior stability, reusability, environmental safety, and cost-effectiveness. The optimal choice is therefore application-dependent, and hybrid strategies combining green reductants with controlled synthesis parameters may offer the best balance of both.

### 3.2 Leaves extracts

**Fig. 2:**
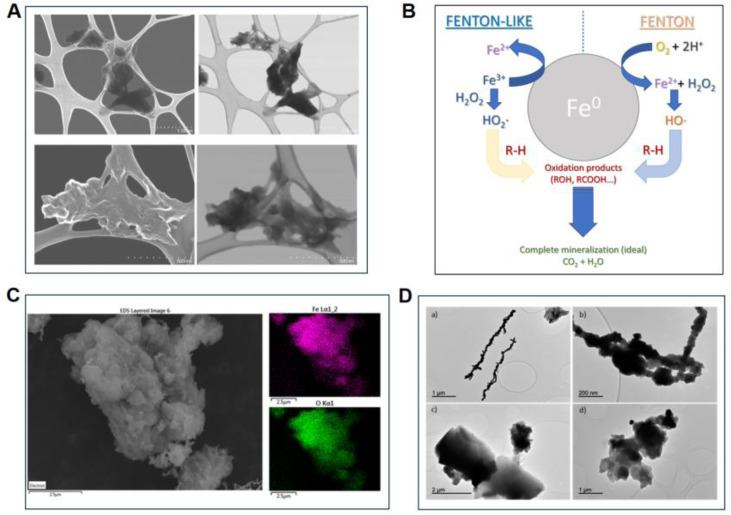
(A) SEM (left) and STEM (right) images of the nZVI at different magnifications and (B) Schematic representation of the Fenton reactions catalyzed by Fe^0^ nanoparticles, this figure has been adapted/reproduced from ref. 82 with permission from MDPI, copyright 2024. (C) SEM images (left) and EDX mapping images (top right: iron and bottom right: oxygen) showing uniform distribution of elements in green-synthesised nZVI and (D) TEM images of nZVI synthesised using NaBH_4_ (a and b) and Tea waste extract (c and d) as the reducing agents, this figure has been adapted/reproduced from ref. 73 with permission from MDPI, copyright 2024.

Leaf extracts have proven effective in the green synthesis of nZVI due to their rich phytochemical composition. For instance, Alexandre-Franco *et al.* employed green tea extract to synthesize nZVI for the advanced oxidation of clinical dyes in simulated solutions.^82^ The experimental results showed that the removal rates of chrysoidine and methylene blue could reach 98.9% and 99.1%, respectively, under the optimal conditions. SEM analysis revealed that the synthesised nZVI formed aggregates with cloud-like and network-like structures, consisting of nanoparticles ranging from a few nanometers to approximately 100 nm in diameter (Fig. 2A). However, aggregation reduces the number of active reaction sites, thereby limiting surface reactivity and catalytic efficiency. In the study by Alexandre-Franco *et al.*, the green tea-derived nZVI operated primarily through a heterogeneous Fenton-like pathway, wherein Fe^0^ was oxidized to Fe^2+^ under aerobic and acidic conditions, generating H_2_O_2_ and subsequently hydroxyl radicals (·OH) capable of mineralizing the target dyes into CO_2_ and H_2_O (Fig. 2B). The polyphenolic capping agents from the tea extract were reported to modulate the surface reactivity of the nanoparticles, influencing both the rate of iron oxidation and the accessibility of active sites for radical generation. In a complementary study, Rodríguez-Rasero *et al.* synthesized nZVI using tea extract *via* the reflux extraction method.^73^ EDX elemental mapping indicated a uniform microscale distribution of iron and oxygen without noticeable aggregation (Fig. 2C). However, the green-synthesized nZVI particles were larger (>500 nm) and exhibited varied morphologies, such as circular and rectangular shapes, compared to the more uniform particles obtained *via* NaBH_4_ reduction (Fig. 2D), further illustrating how the phytochemical composition of the extract influences particle characteristics and, consequently, reactivity.

**Fig. 3:**
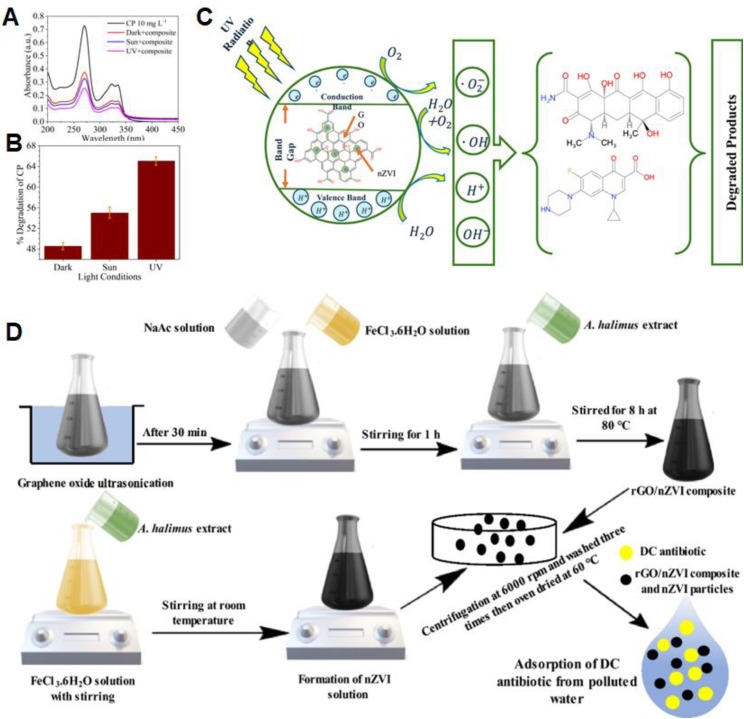
(A) Environmental optimization results for ciprofloxacin degradation using GO-nZVI; (B) efficiency optimization results for the degradation of CP by GO-nZVI particles; (C) proposed mechanism of CP degradation by the GO-nZVI composite, this figure has been adapted/reproduced from ref. 92 with permission from Springer Nature, copyright 2022. (D) Green synthesis of rGO/nZVI composite using *Atriplex halimus* extract and its application for doxycycline removal from water, this figure has been adapted/reproduced from ref. 90 with permission from Elsevier Ltd. (Cell Press), copyright 2024.

Tetracycline and doxycycline contaminate water, threaten ecosystems, and spread antibiotic resistance—urgent removal solutions needed.^91^ Further extending the application of leaf-based biosynthesis, Jha *et al.* developed a graphene oxide-nZVI (GO-nZVI) composite for the removal of tetracycline and ciprofloxacin (CP) from water.^92^ In their approach, sugarcane bagasse was used as a precursor for GO, while Sal leaf extract served as the natural reducing agent. The resulting GO-nZVI composite achieved a 65% removal efficiency of CP under UV light (Fig. 3A and B). The enhanced degradation is attributed to the synergistic action between GO and nZVI. GO provides abundant oxygen-containing functional groups (C=C, C=O, and –OH) that promote antibiotic adsorption, while UV irradiation facilitates charge separation and reactive oxygen species (ROS) generation. The degradation mechanism involves Fe^0^ oxidation to Fe^2+^, H_2_O_2_ formation, and the subsequent generation of ·OH, O_2_˙^−^ and other ROS (Fig. 3C).

In a related study, Abdelfatah *et al.* reported the green synthesis of an rGO/nZVI composite using *Atriplex halimus* leaves extract as both a reducing and stabilizing agent for the removal of doxycycline (DC) antibiotic from water.^90^ GO was first prepared *via* the modified Hummers' method, followed by simultaneous reduction of GO to rGO and iron precursors to nZVI using the plant extract. The resulting rGO/nZVI composite exhibited a DC removal efficiency of 94.6%, outperforming bare nZVI (90%), thereby confirming the synergistic contribution of rGO (Fig. 3D). The enhanced performance was attributed to the improved dispersion and stability of nZVI within the rGO sheets, which increased both the specific surface area and pore volume, facilitating greater interaction with the target antibiotic. Adsorption kinetics followed the pseudo-second-order model and fitted well to the Freundlich isotherm, with a maximum adsorption capacity of 31.61 mg g^−1^ at 25 °C and pH 7. Moreover, the composite retained approximately 60% removal efficiency after six successive regeneration cycles, demonstrating its practical reusability potential.

### 3.3 Seeds extracts

Seed extracts, including those derived from Coffee, Cumin, Thymol, and Ricinus Communis, have been increasingly investigated for the green synthesis of nZVI due to their abundance of phytochemicals and wide availability.^93^ For instance, date seed extract has been combined with FeSO_4_ at elevated temperatures to synthesize stable and well-formed nZVI particles, demonstrating promising characteristics for environmental applications.^94^ Similarly, Moringa Oleifera seed extract has been employed for nZVI synthesis, highlighting the feasibility of using commonly available seeds in scalable, low-cost production methods.^88^

**Fig. 4:**
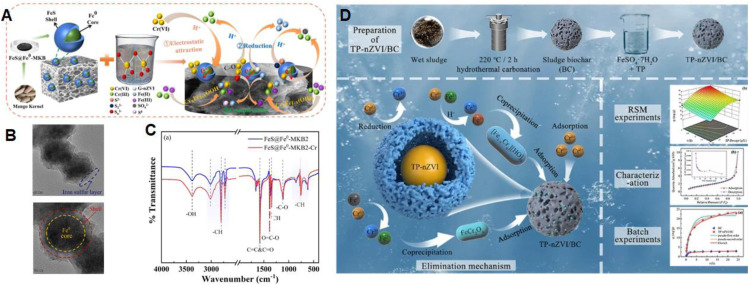
(A) Schematic illustration of the Cr(vi) removal mechanism by FeS@Fe^0^-MKB2 composite; (B) STEM images of FeS@Fe^0^-MKB2; (C) FTIR spectra of FeS@Fe^0^-MKB2 before and after Cr(vi) adsorption, this figure has been adapted/reproduced from ref. 97 with permission from Elsevier Ltd, copyright 2025. (D) Synthesis route and Cr(vi) removal mechanism of tea polyphenol-mediated nZVI-loaded sludge biochar (TP-nZVI/BC), this figure has been adapted/reproduced from ref. 98 with permission from Springer Nature, copyright 2024.

Biochar, a low-cost carbonaceous material, shows great potential in adsorption, nutrient strategies, soil remediation, catalysis, and wastewater treatment applications.^95,96^ In a more advanced application, Tang *et al.* reported the use of mango kernel biochar (MKB) as a support for FeS-modified nZVI (FeS@Fe^0^-MKB) for the removal of hexavalent chromium (Cr(vi)). The composite material exhibited 1.7 times higher Cr(vi) removal efficiency than unmodified nZVI.^97^ The enhanced removal performance was attributed to multiple mechanisms, including electrostatic adsorption, facilitated by the rich functional groups and porous structure of the MKB; reduction reactions, enabled by the strong redox potential of nZVI; co-precipitation and immobilisation, supported by the complexation properties of the corrosion products (Fig. 4A). The core shell structure of FeS@Fe^0^-MKB with Fe^0^ as the core, lamellar iron sulfide (FeS) as the shell, and MKB as the support effectively reduced nanoparticle aggregation and improved reactivity. This architecture enhanced the material's adsorption and reduction capabilities (Fig. 4B and C). Furthermore, FTIR analysis revealed the presence of key functional groups such as –OH, –CH, C–O, C=C, C=O, and O=C–O before and after the reaction. The weakening or shifting of characteristic peaks in the post-reaction material confirmed the active participation of surface functional groups in the Cr(vi) removal process (Fig. 4D).

In a comparable approach, Ma *et al.* prepared a green-modified biochar composite (TP-nZVI/BC) by loading nZVI onto sludge-derived biochar using tea polyphenols (TP) as the green reductant.^98^ The preparation conditions were optimized *via* response surface methodology, identifying TP dosage as the most influential parameter. The resulting TP-nZVI/BC demonstrated a Cr(vi) removal rate 7.6 times greater than that of unmodified biochar, with a maximum adsorption capacity of 105.65 mg g^−1^. Kinetic analysis confirmed that the removal process followed a pseudo-second-order model, indicating chemisorption-dominated uptake. FTIR and XPS analyses revealed that nZVI played a central role in the reductive transformation of Cr(vi) to the less toxic Cr(iii), while the synergistic interplay among surface adsorption, chemical reduction, and co-precipitation collectively enhanced the overall removal performance. These findings corroborate the strategy reported by Tang *et al.* and further highlight the effectiveness of green-synthesized nZVI–biochar composites for hexavalent chromium remediation.

### 3.4 Peels extracts

**Fig. 5:**
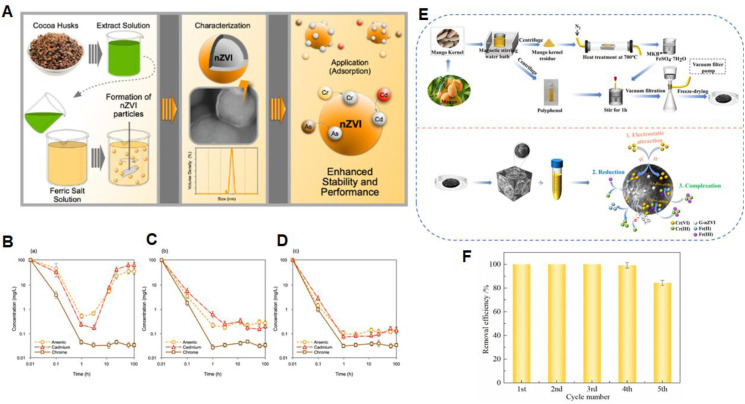
(A) Schematic representation of heavy metal pollutants removal by nZVI; (B) removal efficiency of Cd, Cr, and As by borohydride-reduced nZVI at pH 3.2 over 120 hours; (C) sustained removal efficiency at pH 8.0 by the same method; (D) heavy metal removal by hydrothermally synthesised (HTC) nZVI at pH 3.2 over 120 hours, this figure has been adapted/reproduced from ref. 100 with permission from KeAi Communications Co., Ltd (Elsevier Ltd), copyright 2025. (E) Synthesis route of G-nZVI@MKB from mango kernel waste and proposed Cr(vi) removal mechanism. (F) Regeneration performance of G-nZVI@MKB2 over five successive cycles, this figure has been adapted/reproduced from ref. 101 with permission from Elsevier Ltd, copyright 2024.

The use of fruit peels such as Pomegranate, Mango, Citrus, and Banana offers a cost-effective and sustainable route for the green synthesis of nZVI.^99^ Rashtbari *et al.* demonstrated that pomegranate peel extract effectively reduced iron ions to form nZVI, exemplifying the potential of agricultural waste for environmental nanotechnology applications.^37^ Likewise, Desalegn and Deewan utilized mango peel extract to synthesize nZVI, confirming the dual role of peels as both reducing and stabilizing agents.^72^ Ferro-Falla *et al.* reported the successful green synthesis of nZVI using cocoa husk extracts in combination with hydrothermal carbonization (HTC), achieving efficient removal of heavy metals from simulated solutions.^100^ At an initial concentration of 100 mg L^−1^ for Cd, Cr, and As, the removal rates of nZVI with an average size of 80 ± 30 nm exceeded 98% for As and 99% for Cd and Cr within 1 h at pH 3.2. The nZVI particles, with an average size of 80 ± 30 nm, exhibited over 98% removal efficiency for all three metals within 120 hours under acidic conditions. This high performance was attributed to the material's core–shell structure and carbonaceous framework: the Fe^0^ core provided strong reductive capacity, the FeOOH shell contributed to nanoparticle stability and adsorption, and the carbon-based skeleton helped prevent aggregation and oxidation, thereby enhancing surface reactivity and long-term performance (Fig. 5A–D).

In a related approach, Zhang *et al.* synthesized a novel biochar-supported green nZVI (G-nZVI@MKB) composite by exploiting the “dual identity” of waste mango kernels, where the polyphenol-rich extract served as the green reductant for nZVI synthesis and the lignocellulosic residue was pyrolyzed at 700 °C to produce biochar as the nZVI support (Fig. 5E).^101^ The optimized composite with a Fe/C mass ratio of 2.0 (G-nZVI@MKB2) achieved 99.0% Cr(vi) removal within 360 min under acidic conditions. Kinetic modelling confirmed pseudo-second-order behaviour (*R*^2^ = 0.999), indicating chemisorption-dominated uptake, with a calculated removal capacity of 50.25 mg g^−1^. Comprehensive characterization by XRD, FTIR, SEM, and XPS revealed that the removal mechanism involved three synergistic processes: (1) electrostatic attraction of Cr(vi) anions to the positively charged composite surface, (2) reductive transformation of Cr(vi) to Cr(iii) by Fe^0^ and Fe(ii), and (3) complexation and co-precipitation as Cr_*x*Fe_(1 − *x*)(OH)_3_ and Cr_*x*Fe_(1 − *x*)OOH (Fig. 5E). Furthermore, the composite exhibited excellent reusability, maintaining complete Cr(vi) removal during the first three regeneration cycles and retaining 83.6% removal efficiency after five successive cycles (Fig. 5F), confirming its practical potential for Cr(vi)-contaminated wastewater treatment.

### 3.5 Other plant components

In addition to leaves, seeds, and peels, other plant components such as stems, barks, and flowers have also been employed for the green synthesis of nZVI.^40,102^ For instance, Machado *et al.* utilized various citrus fruit wastes, including the peel, albedo, and pulp, to synthesize nZVI, with lemon waste extract exhibiting the highest reactivity.^103^ Dhiss *et al.* demonstrated the successful synthesis of nZVI using palm petiole extract, showcasing the valorization of locally available agricultural waste.^15^

**Fig. 6:**
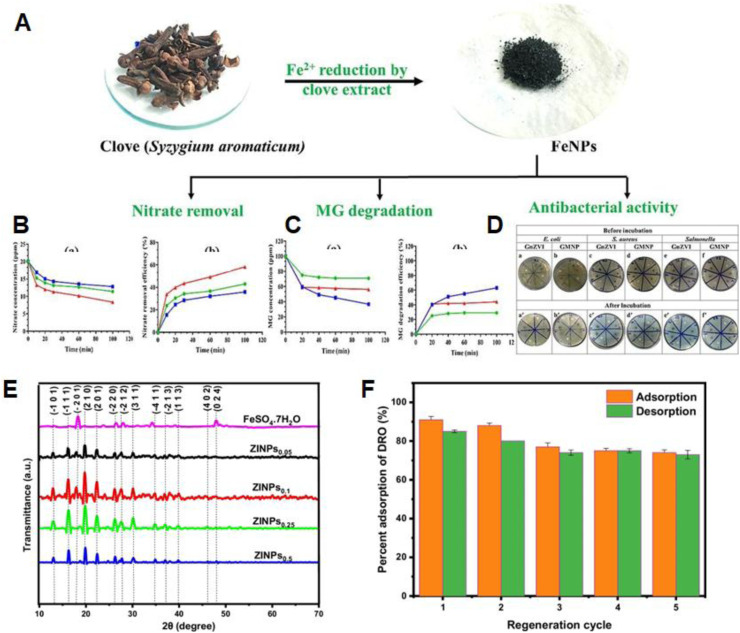
(A) Schematic representation of nZVI synthesis using clove extract for pollutant removal applications; (B) nitrate concentration reduction and corresponding removal efficiencies by SA-FeNPs, SA-MNPs, and chemically synthesised nZVI (ZVIPs); (C) MG concentration reduction and degradation efficiency by SA-FeNPs, SA-MNPs and ZVIPs; (D) (a–f) zone inhibition of *E. coli*, *S. aureus*, and *S. enterica* before incubation, and (a′–f′) zone inhibition of *E. coli*, *S. aureus* and *S. enterica* after incubation of SA-FeNPs and SA-MNPs, this figure has been adapted/reproduced from ref. 104 with permission from Elsevier Ltd, copyright 2024. (E) XRD patterns of FeSO_4_·7H_2_O and ZINPs synthesized at different precursor concentrations (0.05–0.5 M) using *Moringa oleifera* leaf extract. (F) Adsorption–desorption regeneration cycles of ZINPs for DRO removal over five successive cycles, this figure has been adapted/reproduced from ref. 105 with permission from Springer Nature, copyright 2023.

Gaminda *et al.* employed clove extract as a green alternative to conventional reductants like NaBH_4_ and KBH_4_ for the synthesis of iron-based nanoparticles, including zerovalent iron (SA-FeNPs) and magnetite particles (SA-MNPs) (Fig. 6A).^104^ In batch experiments, nitrate (NO_3_^−^) removal was evaluated over time, with nZVI, SA-FeNPs, and SA-MNPs achieving removal efficiencies of 58.3%, 43%, and 36%, respectively, within 100 min (Fig. 6B). For malachite green (MG) degradation, SA-MNPs showed superior efficiency (63%) compared to nZVI (44%) and SA-FeNPs (29%) (Fig. 6C). Furthermore, the green-synthesized nanoparticles exhibited significant antibacterial activity against both Gram-positive and Gram-negative bacterial strains, as demonstrated by the zones of inhibition (Fig. 6D).

Extending the application of *Moringa oleifera* as a green reducing agent, Ubah *et al.* synthesized zerovalent iron nanoparticles (ZINPs) using *M. oleifera* leaf extract for the adsorptive removal of diesel range organics (DRO) from contaminated water.^105^ The synthesized ZINPs exhibited quasi-nanospherical and nanocubic morphologies with an average particle diameter of ∼50.9 nm and a crystallite size of 15.31 nm. XRD analysis confirmed the crystalline nature of the ZINPs at various precursor concentrations (0.05–0.5 M), with characteristic diffraction peaks corresponding to Fe^0^, iron oxide phases (FeO, Fe_2_O_3_, Fe_3_O_4_), and γ-FeOOH (lepidocrocite), indicating partial surface oxidation of the nZVI (Fig. 6E). Process optimization using response surface methodology (RSM) revealed a maximum DRO removal efficiency of 92.6% under optimized conditions (pH 8, 25 °C, 2 g L^−1^ dosage, 8 h contact time). The adsorption equilibrium was best described by the Langmuir isotherm model, with a maximum monolayer adsorption capacity of 7.194 mg g^−1^, while kinetic analysis followed the pseudo-second-order model, suggesting chemisorption as the dominant mechanism. Moreover, the reusability study demonstrated that ZINPs retained approximately 74% DRO adsorption efficiency after five successive adsorption–desorption regeneration cycles using HCl as eluent, confirming the practical recyclability and stability of the nanosorbent (Fig. 6F). Other plant-derived materials have also shown promise: flaxseed gum and Piliostigma thonningii flower extracts have been reported to yield nZVI with enhanced dispersion, stability, and reactivity.^106,107^

Different parts of plants have been utilized as reducing and stabilizing agents in the green synthesis of nZVI. Each plant part contains unique phytochemicals that influence the size, stability, and reactivity of the produced nanoparticles. Table 2 summarizes the main types of plant parts used.

**Table 2:** Comparison of different plant parts used in green synthesis of nZVI

Plant part	Main phytochemicals	Advantages	Drawbacks	Typical examples (plants/studies)	Ref.
Leaves	Polyphenols, flavonoids, sugars, proteins	High reducing and capping ability; produces small, stable nanoparticles; easy extraction	Requires large biomass; seasonal variation in composition	*Moringa oleifera*, *Azadirachta indica*, *Camellia sinensis*	108 and 109
Roots	Alkaloids, saponins, tannins	Provides good stability and moderates reducing potential	Lower yield; limited availability for large-scale use	*Withania somnifera*, *Glycyrrhiza glabra*	110
Stems	Phenolics, lignin, cellulose	Good stabilizing matrix; supports uniform particle dispersion	Fewer active biomolecules; difficult extraction	*Tinospora cordifolia*, *Bacopa monnieri*	110
Seeds	Oils, proteins, carbohydrates	Abundant and renewable values agricultural by-products	Composition varies; may produce larger or less uniform particles	*Vigna radiata*, *Coffea arabica*	110
Peels (fruit/vegetable)	Polyphenols, citric acid, ascorbic acid	Low-cost waste material; rich in antioxidants; environmentally friendly	Variable composition: Impurities may affect nanoparticle quality	*Citrus sinensis*, *Musa paradisiaca*, *Punica granatum*	109 and 110

Notably, contradictory performance trends are reported for leaf-extract-synthesized nZVI. While some studies report removal efficiencies exceeding 95% for dyes and antibiotics, others observe significantly reduced kinetics compared to chemically synthesized counterparts. These discrepancies can be attributed to variations in extract concentration, polyphenol composition, extraction solvent, and Fe precursor speciation, which strongly influence nucleation rates and surface passivation. Excessive phytochemical capping may inhibit electron transfer by blocking Fe^0^ active sites, explaining why higher phenolic content does not always correlate with higher reactivity.

## 4. Supporting materials for nZVI

The incorporation of supporting materials has emerged as a crucial strategy to enhance the dispersion stability, and reactivity of nZVI. Supported nZVI systems improve the distribution of Fe^0^ particles and facilitate the adsorption of contaminants prior to degradation, thus boosting the overall efficiency of water treatment process.^86^ A wide range of supporting materials including clays, magnetic substrates, carbon-based materials, zeolites, MOFs, COFs, biochar, and polymers has been extensively explored for their ability to interact synergistically with nZVI.^111^ These materials enhance nZVI dispersion, stability, and efficiency for various environmental and biological applications. This review focuses on clay minerals, carbon-based materials, and inorganic supports due to their availability, low cost, and proven effectiveness in improving green-synthesized nZVI systems.

### 4.1 Clay supports

Clay materials such as bentonite, sepiolite, montmorillonite, and kaolin offer high surface areas, cation exchange capacities, and porosity, making them ideal candidates for supporting nZVI.^40^ These materials enhance the stability and dispersibility of iron nanoparticles, preventing rapid aggregation and oxidation.^112,113^ For instance, Hassan *et al.* synthesized bentonite-supported nZVI using leaf extract, obtaining a stable Fe:bentonite mass ratio of 0.3 : 0.7 and a pollutant adsorption capacity 45 times greater than that of natural bentonite.^114^ Similarly, nZVI composites supported on sepiolite, montmorillonite, and kaolin and synthesized *via* green methods have shown improved reactivity due to their porous networks and abundant functional groups.^115,116^ Dhiss *et al.* also reported the effective degradation of pollutants using palm petiole extract to synthesize nZVI supported on El Hamma bentonite, highlighting the valorization of local clay resources.^40^

### 4.2 Carbon supports

Carbonaceous materials are widely used as support for nZVI due to their high specific surface area, strong electron conductivity, and ability to facilitate electron transfer and adsorption of contaminants.^86,117^ For instance, Rashtbari *et al.* synthesized nZVI on activated carbon using Pomegranate Peel extract, creating an AC/nZVI composite that exhibited enhanced adsorption and catalytic degradation of pollutants.^37^ Biochar, a porous carbonaceous material derived from biomass pyrolysis, has also proven to be a promising support material for nZVI. For example, nZVI was synthesized on oak wood biochar using tea polyphenol extract, resulting in a composite with improved environmental stability and reactivity^118^

**Fig. 7:**
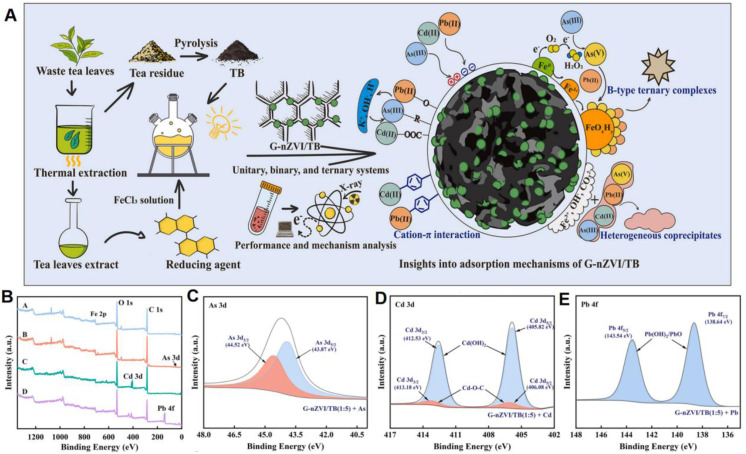
(A) Schematic illustration of synthesis of G-nZVI/TB and proposed mechanisms of metal(loid) removal; (B) XPS spectra of G-nZVI/TB (1 : 5) before and after reaction with As(iii), Cd(ii), and Pb(ii) in single-metal systems; (C–E) XPS high-resolution spectra showing the binding energies of (C) As 3d before and after As(iii) oxidation, (D) Cd 3d confirming Cd species precipitation, and (E) Pb 4f revealing Pb complexation and precipitation products, this figure has been adapted/reproduced from ref. 119 with permission from Elsevier Ltd, copyright 2022.

Wang *et al.* developed a novel green-synthesized nZVI–tea biochar composite (G-nZVI/TB) using waste tea leaves both as the reducing agent and as the pyrolytic precursor for the biochar support.^119^ The composite was tested for its ability to remove single and mixed heavy metals from aqueous solutions. The results revealed that metal(loid) removal occurred *via* multiple mechanisms, including electrostatic adsorption, ion exchange, co-precipitation, cation-π interactions, oxidation-complexation, and B-type ternary complexation (Fig. 7A). X-ray photoelectron spectroscopy (XPS) was employed to monitor surface chemical changes. Before treatment, characteristic peaks of Fe 2p, O 1s, and C 1s were observed, confirming the presence of key elements. After reaction with As(iii), Cd(ii), and Pb(ii), additional peaks (As 3d, Cd 3d, Pb 4f) were detected, indicating successful adsorption of heavy metals (Fig. 7B). Notably, As(iii) oxidation to As(v) was evident from the shift in the As 3d binding energies (Fig. 7C), while Cd^2+^ and Pb^2+^ were shown to form precipitates and hydroxide complexes such as CdCO_3_, Cd(OH)_2_, PbO·PbCO_3_, and Pb(OH)_2_ (Fig. 7D and E).

**Fig. 8:**
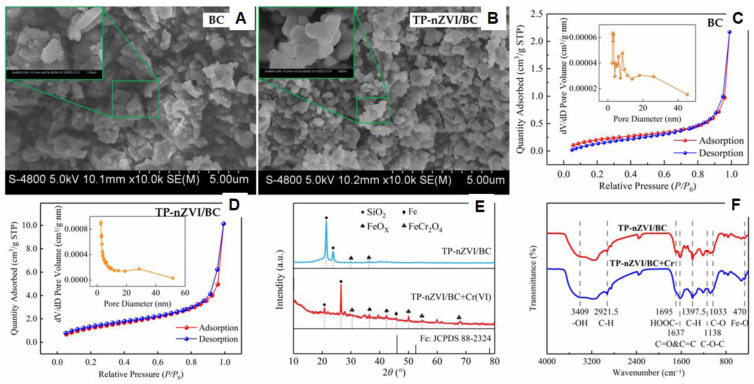
(A and B) SEM images of BC and TP-nZVI/BC composite; (C and D) nitrogen adsorption–desorption isotherms from BET analysis for BC and TP-nZVI/BC; (E) XRD spectra of TP-nZVI/BC before and after Cr(vi) treatment; (F) FTIR spectra before and after Cr(vi) adsorption, this figure has been adapted/reproduced from ref. 36 with permission from MDPI, copyright 2025.

Zhao *et al.* successfully synthesized a green composite by modifying sludge-derived biochar (BC) with tea polyphenols (TP) and loading nZVI onto it (TP-nZVI/BC) for the removal of Cr(vi) from wastewater.^36^ The synergistic interaction between nZVI and biochar played a crucial role in enhancing the removal efficiency. Scanning electron microscopy (SEM) revealed a rough and porous structure on the BC surface (Fig. 8A), with nZVI particles uniformly distributed across the surface and within the pores of the TP-nZVI/BC composite (Fig. 8B). The agglomerated nature of nZVI confirmed its successful incorporation into the biochar matrix. Fourier-transform infrared (FTIR) spectroscopy detected new Fe–O characteristic peaks post-synthesis, confirming the formation of iron oxides during the process. Brunauer–Emmett–Teller (BET) analysis revealed that both BC and TP-nZVI/BC composites contained micropores and mesopores. The nitrogen adsorption–desorption isotherms exhibited hysteresis loops near *P*/*P*_0_ = 1 for BC (Fig. 8C) and a rapid increase in adsorption at *P*/*P*_0_ > 0.8 for TP-nZVI/BC (Fig. 8D), suggesting enhanced mesoporosity and adsorption capacity. X-ray diffraction (XRD) analysis confirmed the amorphous nature of the composite and the presence of Fe^0^, with additional peaks (FeCr_2_O_4_ and FeO_*x*_) appearing after Cr(vi) treatment, indicating reduction to Cr(iii) and surface deposition (Fig. 8E). FTIR spectra before and after adsorption showed a weakening of functional group intensities such as –OH, C=O/C=C, –COOH, and C–H demonstrating their participation in Cr(vi) removal reactions (Fig. 8F). In addition, pyrogenic carbon (PC) derived from pinewood treated with hematite has also been employed to support nZVI. The resulting nZVI/PC composites displayed superior performance due to increased surface area and reactivity, confirming the potential of biochar-based supports in enhancing pollutant remediation.^86^

### 4.3 Bimetallic nanoparticles

**Fig. 9:**
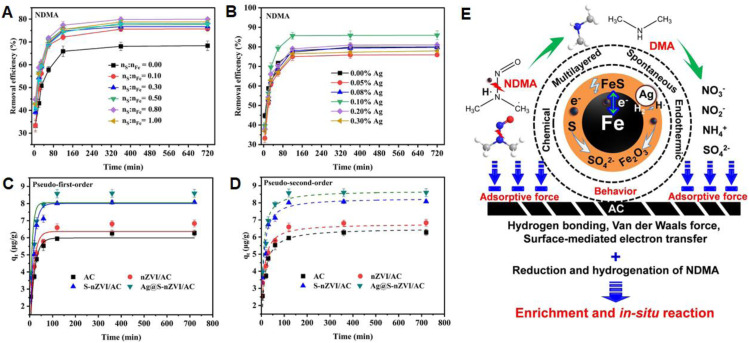
(A and B) NDMA removal efficiency by Ag@S-nZVI/AC under varying Ag content and nS:nFe ratios; (C and D) pseudo-first-order (PFO) and pseudo-second-order (PSO) kinetic fitting for NDMA adsorption on various composites; (E) schematic illustration of interfacial interactions and degradation pathways of NDMA on Ag@S-nZVI/AC, this figure has been adapted/reproduced from ref. 129 with permission from Elsevier Ltd, copyright 2025.

Bimetallic nanoparticles (BNPs) outperform monometallic ones due to synergistic effects between the two metals, resulting in improved catalytic, reductive, and adsorption properties for environmental remediation.^67^ The effectiveness of bimetallic nanoparticles (BNPs) is further amplified through the incorporation of support materials, which help stabilize the nanoparticles, prevent agglomeration, and improve catalytic performance.^120^ For example, Lin *et al.* synthesized Fe/Ni BNPs using Eucalyptus leaf extract, serving as both reducing and stabilizing agents. The resulting calcined product exhibited high stability and reactivity for the degradation of chlorinated organic compounds.^121^ Zhu *et al.* prepared Fe/Cu BNPs using Green Tea extract and vacuum-dried the composite, obtaining nanoparticles with significantly enhanced catalytic efficiency.^122^ Likewise, Ndagijmana *et al.* utilized Punica granatum (pomegranate peel) extract to synthesize Fe/Ag core–shell BNPs *via* the reduction of silver nitrate in an iron chloride solution. Li *et al.* synthesized a green composite (GT-BC@nZVI/Cu) by combining green tea extract, biochar (BC), and copper salts.^123^ The resulting material exhibited excellent adsorption and degradation performance for aqueous contaminants.^124^ Other supports, such as silty clay and natural polymers, have also proven effective in enhancing the dispersion and reactivity of BNPs in various environmental contexts.^125,126^ The synthesis approach whether through chemical reduction, co-precipitation, or green routes plays a crucial role in determining the physicochemical characteristics and pollutant removal capabilities of BNPs.^127,128^ In a notable example, Riaz Ahmad *et al.* developed an activated carbon-supported bimetallic composite coated with sulfur and silver (Ag@S-nZVI/AC) for the effective removal of *N*-nitrosodimethylamine (NDMA).^129^ The composite with 0.1% Ag and nS:nFe ratio of 0.80 achieved the highest NDMA removal, reaching equilibrium within 360 minutes (Fig. 9a and b). Kinetic modelling showed that the pseudo-second order (PSO) model best described the NDMA adsorption process (Fig. 9c and d). NDMA removal occurred through several mechanisms: (i) multilayer chemical adsorption, mediated by hydrogen bonding and van der Waals forces on the AC surface; (ii) redox reactions, *via* surface-mediated electron transfer on Fe^0^, FeS, and S; (iii) hydrogenation, involving cleavage of the N–N bond in NDMA, producing end-products such as dimethylamine (DMA), SO_4_^2−^, NO_3_^−^, NO_2_^−^, and NH_4_^+^(Fig. 9e).

These BNPs demonstrated superior antimicrobial and pollutant degradation activities compared to their monometallic counterparts.^130^ The interactions between two metals in BNPs result in enhanced catalytic and reductive capabilities, as exemplified by Fe/Ni BNPs, which exhibit superior electron transfer efficiency and accelerated degradation of organic pollutants. Furthermore, the bimetallic configuration promotes the formation of unique crystal facets and increases the number of reactive sites, thereby improving adsorption capacity and reaction kinetics, both essential parameters for effective contaminant removal. The presence of a secondary metal also enhances nanoparticle stability and dispersion, reducing agglomeration and improving their performance and longevity in aqueous environments.

Different supporting materials for nZVI have been developed to enhance its dispersibility, stability and removal efficiency of contaminants. These carriers vary in composition (*e.g.*, clays, carbon-based materials, inorganic supports) and each offers distinct advantages depending on the target application and environmental conditions. Table 3 summarizes the main types of supporting materials, their advantages, drawbacks and typical applications.

**Table 3:** Comparison of different supporting materials for nZVI

Supporting material	Advantages	Drawbacks	Applications	Ref.
Clay minerals	Low cost, high adsorption capacity, good dispersion of nZVI on clay layers	May reduce reactive sites of nZVI, possible slower kinetics	Groundwater/soil remediation of arsenic, heavy metals	131
Carbon-based materials	High surface area, good conductivity, enhances reduction + adsorption	Cost may be higher, potential for secondary pollution if not stable	Removal of Cr(vi), organics from water	132
Inorganic supports	Improved stability of nZVI, reduced agglomeration, good mechanical strength	Possibly higher cost, some supports may block access to nZVI core	Heavy metal removal, Ni^2+^, U(vi) remediation	133

## 5. Surface modification of nZVI

Surface modification of nZVI significantly enhances its reactivity, stability, and mobility, while also reducing particle aggregation and improving dispersibility in aqueous environments (Table 4). Common modification techniques include surface coatings method, supporting method, sulfidation method and bimetallic method.

**Table 4:** Summary table comparing reactivity, stability, and ageing resistance for each modification strategy

Modification techniques	Sythesis methods/conditions	Reactivity	Stability	Ageing resistance	Ref.
Surface coatings method	Polymeric, stabilizers, surfactants, or other stabilizing agents	Surface coatings will create spatial and electrostatic barriers, effectively reducing interparticle magnetic attraction and preventing agglomeration and oxidation; besides, surface coatings will also broaden its operational pH range, and reduces biotoxicity	Aqueous dispersibility is improved	The ageing resistance is improved, and the coating layer can partially isolate water and oxygen, and slow down the oxidation passivation of Fe core	61
Supporting method	Porous substrates	Improve nZVI's pecific surface area and adsorption capacity	Aqueous dispersibility and material recyclability are enhanced	The ageing resistance is improved, the carrier provides a “refuge” for nZVI, which shields the water and oxygen erosion in the environment to some extent	59
Sulfidation method	Sulfide treatment to form a conductive FeS_*x*_ shell around the nanoparticles	Improve electron conductivity and inhibits side reactions with water and oxygen; enhance antioxidant capacity	Physical stability is improved, and magnetic agglomeration is reduced	The ageing resistance are greatly improved, and the FeS shell can effectively block water molecules and oxygen, greatly delaying the corrosion and passivation of Fe in the core	130
Bimetallic method	Secondary metal (*e.g.*, Pt, Pd, Cu, Ni, and Ti)	Enhance electron transfer efficiency and reaction performance, prevent surface passivation	Material stability is improved	The ageing resistanceare seriously reduced. The galvanic effect accelerates the degradation of pollutants and the consumption of nZVI itself, which leads to the rapid inactivation of materials and poor anti-aging ability	134

**Fig. 10:**
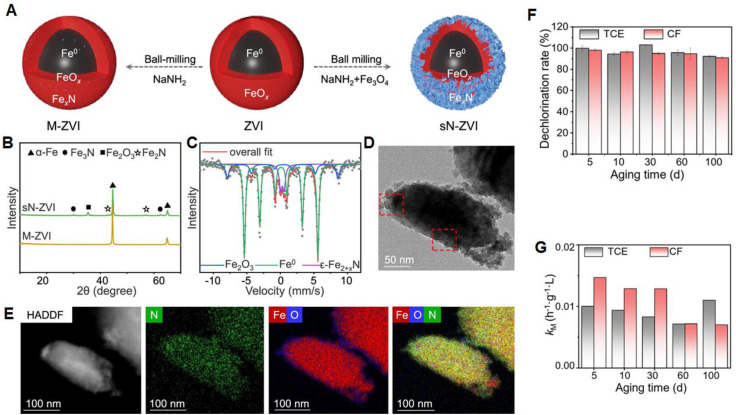
(A) Schematic illustration of the synthesis of sN-ZVI; (B) XRD patterns of sN-ZVI; (C) ^57^Fe Mössbauer spectrum of sN-ZVI recorded at 13 K; (D) HRTEM images of sN-ZVI; (E) HAADF image of elemental distributions of sN-ZVI; (F) Mass balance of chloride (based on the measurement of chloride anion); (G) mass-normalised rate constants (*k*_M_) (units: h^−1^ g^−1^ L) for TCE and CF dechlorination by sN-ZVI at different ageing times, this figure has been adapted/reproduced from ref. 135 with permission from American Chemical Society, copyright 2025.

Li *et al.*^135^ developed surface-nitrided ZVI (sN-ZVI) using a mechanochemical molten-salt-assisted method for the removal of trichloroethylene (TCE) and chloroform (CF) (Fig. 10a). XRD analysis revealed that M-ZVI lacked the FexN peaks present in nZVI, indicating that Fe_3_O_4_ facilitated the nitridation process (Fig. 10b). The ^57^Fe Mössbauer spectrum of nZVI displayed two sextets (α-Fe_2_O_3_ and Fe^0^) and one doublet (ε-Fe^2+*x*^N, 0 ≤ *x* ≤ 1), confirming successful surface nitridation while retaining Fe^0^ content, crucial for promoting electron transfer (Fig. 10c). High-resolution TEM images showed lattice spacings of 2.98 Å and 1.62 Å, corresponding to the (101) plane of ε-Fe_3_N and the 
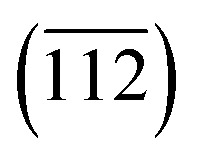
 plane of ε-Fe_2_N, respectively (Fig. 10d), indicating the formation of distinct Fe–N complexes. Additionally, surface nitridation reduced the shell thickness of ZVI particles (Fig. 10e), likely due to the space confinement effect in the molten NaNH_2_–NaOH medium. Impressively, after 100 days of ageing in aqueous conditions, nZVI maintained complete dichlorination of TCE and CF, with only a slight decline in reaction rates (Fig. 10f and g).

**Fig. 11:**
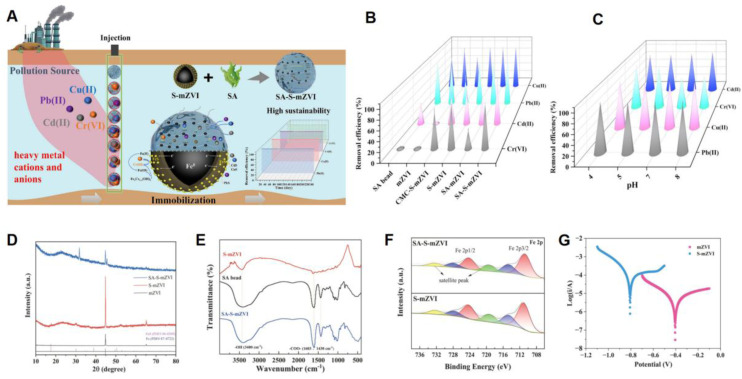
(A) The removal mechanism of SA-S-mZVI for heavy metal cations and anions; The removal efficiency of different materials; (B) and effect of pH (C) for Cr(vi), Cd(ii), Pb(ii), and Cu(ii); (D) XRD of S-mZVI and SA-S-mZVI; (E) FTIR spectra of SA bead, S-mZVI, and SA-S-mZVI; (F) High-resolution XPS spectrum of Fe 2p; (G) Tafel curves of mZVI and S-mZVI, this figure has been adapted/reproduced from ref. 138. with permission from Elsevier Ltd, copyright 2025.

Recent studies have highlighted that heavy metal contamination in water poses serious environmental and health risks worldwide, demanding urgent remediation.^136,137^ Therefore, Wang *et al.*^138^ synthesized a green sulfidated micro-ZVI-based hydrogel (SA-S-mZVI) using sodium alginate (SA) as a biomass-derived matrix for the simultaneous removal of cationic (Cu^2+^, Pb^2+^, Cd^2+^) and anionic (Cr(vi)) heavy metals from groundwater. The removal mechanisms included electrostatic attraction, ion exchange, and complexation (Fig. 11A). Long-term column experiments (240 days) demonstrated exceptional removal efficiencies exceeding 99.9% for all four heavy metals. The synergistic combination of SA and S-mZVI contributed to improved performance: SA, rich in –OH and –COOH groups, enhanced adsorption while suppressing aggregation and improving the dispersion of S-mZVI particles (Fig. 11B). The material was also effective over a wide pH range (4–8) (Fig. 11C). XRD analysis confirmed that the SA coating did not alter the crystalline structure of S-mZVI but reduced the intensity of characteristic Fe^0^ and FeS peaks (Fig. 11D). FTIR spectra showed characteristic peaks of SA at 3400, 1603, and 1430 cm^−1^, which were also present in SA-S-mZVI but with slight shifts, verifying the successful surface modification (Fig. 11E). High-resolution XPS analysis (Fe 2p) revealed the absence of Fe^0^ peaks, indicating encapsulation by iron oxide or sulfide layers. The reduced Fe(ii) content in SA-S-mZVI compared to S-mZVI suggested partial oxidation of Fe^0^ during the coating process (Fig. 11F). Electrochemical Tafel analysis showed that sulfidation reduced the corrosion potential of nZVI, implying improved electron transfer and enhanced reduction capacity (Fig. 11G).

**Fig. 12:**
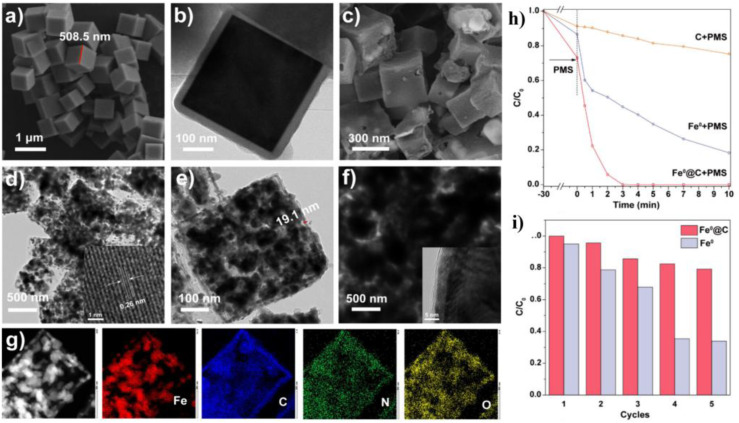
(a) SEM image of PB; (b) TEM image of PB@PDA; (b) SEM image of Fe^0^@C nanocube; (d and e) HRTEM images of Fe^0^@C nanocube; (f) HRTEM image of Fe^0^ nanoparticles; (g) EDX-elemental mapping images of Fe^0^@C nanocube; (h) removal of BPA by different systems conditions; (i) cycling measurements for BPA degradation over Fe^0^@C nanocube, this figure has been adapted/reproduced from ref. 139 with permission from American Chemical Society, copyright 2025.

Similarly, Li *et al.*^139^ designed Fe^0^@C nanocubes to activate peroxymonosulfate (PMS) for efficient degradation of bisphenol A (BPA). The precursor Prussian Blue (PB) nanocubes had smooth surfaces and uniform particle sizes (∼500 nm) (Fig. 12A), while polydopamine (PDA) coating preserved their cubic structure during synthesis (Fig. 12B). Post-calcination, the Fe^0^@C nanocubes maintained a porous carbon-encapsulated structure, promoting both mass diffusion and pollutant adsorption (Fig. 12C and E). In contrast, directly calcined PB cubes without PDA coating resulted in aggregated Fe^0^ nanoparticles with oxide layers, leading to passivation and reduced reactivity (Fig. 12F). Elemental mapping showed uniform distribution of Fe, C, N, and O in the Fe^0^ @C nanostructure (Fig. 12G). The Fe^0^@C nanocubes exhibited superior BPA degradation performance, achieving complete removal within 5 minutes, outperforming both uncoated Fe^0^ and carbon materials alone. This efficiency was attributed to the protective carbon layer, which inhibited Fe^0^ aggregation and dissolution while providing additional reactive sites and maintaining structural integrity (Fig. 12H). The nanocubes also demonstrated excellent reusability and stability over multiple cycles (Fig. 12I).

## 6. Green synthesis and support materials in nZVI environmental applications

Each of these synthesis strategies distinctly influences the particle size, structural integrity, and reactivity of nZVI, thereby affecting its performance in environmental remediation applications.^70,134,140^ In addition to these conventional techniques, green synthesis methods have gained increasing attention for their sustainability and environmental safety. Among them, plant-mediated synthesis is the most widely adopted, where phytochemicals such as polyphenols, flavonoids, and sugars act as natural reducing and capping agents to convert Fe^3+^ or Fe^2+^ ions into Fe^0^ nanoparticles under mild conditions.^108^ This route eliminates the need for hazardous reagents and allows better control over particle morphology and dispersion.

Furthermore, microbial and hybrid green–chemical methods have recently been explored to enhance reaction kinetics and nanoparticle uniformity.^110^ In these processes, microorganisms or natural extracts work synergistically with mild chemical reducers, yielding biocompatible and highly reactive nZVI. Each approach presents distinct benefits in terms of cost, scalability, and reactivity; however, challenges such as particle aggregation, variable extract composition, and limited standardization remain [130]. Future optimization of these eco-friendly synthesis routes will be essential to balance performance, reproducibility, and sustainability in large-scale applications.^141–143^ nZVI has demonstrated excellent efficiency in degrading a wide range of pollutants, including: heavy metals,^15^ organic pollutants like pesticides,^54^ dyes,^40^ phenols,^43^ and pharmaceuticals.^41^

In aqueous environments, nZVI facilitates reduction, degradation, or immobilisation of contaminants through redox reactions and adsorption processes. Additionally, nZVI has been successfully applied in permeable reactive barriers (PRBs) to intercept and treat contaminated groundwater and in soil remediation, where it prevents the leaching and bioaccumulation of toxic substances by immobilising them.^144^ Thanks to its high reactivity and surface area, nZVI is also utilised as a catalyst in various advanced oxidation processes, further enhancing its role in environmental clean-up.^145^ The environmental uses of nZVI, which is produced by various plant components, are listed in Table 5.

**Table 5:** Characteristics, synthesis conditions, and environmental remediation performance of plant-synthesised nZVI and nZVI-supported materials

Plants (part)	Type of extract/support material	Synthesis conditions/material type	Size (nm)	Morphology	Stability	Environmental application	Removal capacity & reaction time	Ref.
*Punica granatum* peel	Ethanol; quartz sand (as support in some studies)	Peel extraction + FeCl_3_ + PbSO_4_	48	Spherical	—	Removal of ciprofloxacin and Cu(ii)	99%, 70 min	146
*Ricinus communis* seed	Distilled water	Seed extracts + Fe^3+^ ions	20	Spherical	Good stability	Adsorption of methylene blue	—	68
Green tea and eucalyptus leaves	Deionized water; porous-activated carbon/sulfate (as support in some studies)	Leaf extracts + FeSO_4_	20–80	Quasi-spherical	Strong stability	Removal of nitrate nitrogen	93%, 95 min	147
Coffee	–; Kaolinite clay (support)	—	—	—	—	Orange(II) removal	60%, 15 min	148
Pomegranate rind	–; Bentonite clay/Cu(ii) (support)	—	—	—	—	Removal of tetracycline	72%	149
*Mentha spicata* leaf	Chitosan (support/matrix)	—	—	—	—	Removal of As(iii) and As(v) from aqueous solution	99.65%, 30 min and 98.7%, 30 min	150
Pomegranate peel	Trametes suaveolens biochar (support)	—	—	—	—	Cr(vi) removal	100%, 90 min	151
Eucalyptus leaves	–; Bentonite clay (support)	—	—	—	—	Catalytic reduction of 4-nitrophenol	95%, 3 h	152
Waste palm petiole	–; El Hamma bentonite clay (support)	—	—	—	—	Heterogeneous Fenton of Crocein Orange G dye	100%, 3 h	153
*Myrtus communis* leaf	Methanol	Extracts + NaOH + ascorbic acid + FeCl_3_	40–60	Spherical	Good stability	—	—	62
*Salvia officinalis* leaf	Milli-Q water	Leaf extraction + FeCl_3_	5–25	Spherical	Good stability	Removal of cationic dye ethyl violet	—	154

Several studies have demonstrated that moderate doses of nZVI stimulate microbial activity, enhance dichlorination kinetics, and improve the resilience of microbial communities under oxidative stress. However, excessive nanoparticle loading may inhibit microbial growth due to localized pH shifts or iron oxide accumulation. Therefore, controlling nZVI concentration and maintaining a balanced abiotic–biotic synergy are critical for achieving sustainable combined remediation.

In green-synthesised nZVI, biomolecules originating from plant extracts (polyphenols, proteins, organic acids) serve dual roles as reducing and capping agents, influencing electron density and surface charge. These molecules stabilize Fe^0^ nuclei, slow oxidation, and modulate electron transfer between the metal core and contaminants. Consequently, the mechanistic behaviour of green nZVI is often distinguished by enhanced surface reactivity and controlled electron release, providing both efficiency and selectivity in pollutant removal.According to published research, nZVI were produced from plants that have been stabilized using various substances and used to remove pollution from the environment. Table 5 illustrates the environmental treatment by nZVI-supported materials.

The data presented in Table 5 provide a comparative summary of the main studies on green synthesis and supported forms of nZVI. As shown in Table 5, several studies demonstrated that the type of plant extract strongly affects nanoparticle size, morphology, and stability. Extracts rich in phenolic and flavonoid compounds, such as those derived from Moringa oleifera, Azadirachta indica, and Camellia sinensis, resulted in smaller and more stable nZVI particles with higher reactivity. This improvement is mainly attributed to the natural reducing and capping agents in these extracts, which control nucleation and inhibit particle aggregation, as reported in the corresponding references listed in Table 1. Meanwhile, Table 3 highlights the influence of different supporting materials on the physicochemical behaviour of nZVI. Biochar and clay support generally enhance the dispersion and surface reactivity of the nanoparticles compared to unmodified nZVI. The studies cited in Table 3 indicate that these supports prevent particle agglomeration, increase surface area, and prolong the lifetime of reactive iron species, leading to higher removal efficiencies for heavy metals and organic contaminants. In general, the data summarised in both Tables 3 and 5 confirm that the synthesis route, biological source, and supporting matrix collectively determine the catalytic performance, stability, and environmental compatibility of green-synthesised nZVI.

## 7. Recent catalytic and adsorptive applications of nZVI-based materials

In recent years, the development of nZVI-based materials has expanded significantly beyond traditional remediation, particularly in advanced oxidation processes (AOPs) and adsorption technologies. These multifunctional materials demonstrate high surface reactivity and tunable properties, making them ideal for catalysis and sorption applications across various environmental contexts. Table 6 summarizes selected recent applications of nZVI catalysts used in AOPs for the removal of diverse pollutants, including dyes, pharmaceutical residues, heavy metals, and industrial chemicals. These systems operate under carefully optimized conditions (*e.g.* pH, temperature, catalyst dosage), employing oxidative or reductive mechanisms to generate highly reactive species such as ˙OH radicals, SO_4_˙^−^ radicals, O_2_˙^−^ anions, or other ROS. The efficiency of pollutant degradation, recyclability of catalysts, and the nature of the active species are highlighted to demonstrate the versatility of these catalytic systems in environmental remediation. In parallel, Table 7 presents a compilation of recent studies utilizing nZVI-based adsorbents for the removal of various inorganic and organic contaminants. The adsorption performance is characterized by parameters such as maximum adsorption capacity (*Q*_max_), adsorption isotherms (Langmuir or Freundlich), sorption mechanisms (physisorption or chemisorption), kinetic models (*e.g.* PSO, PFO), and thermodynamic behaviour (endothermic or exothermic). These metrics provide insight into the efficiency and mechanisms of interaction between the adsorbents and target pollutants, guiding the design of tailored adsorptive systems. Together, these tables reinforce the strategic role of engineered nZVI-based materials in sustainable environmental technologies and underscore their growing importance in advanced water treatment and pollution control systems.

**Table 6:** Recent applications of nZVI catalysts for AOPs

Catalyst	Pollutants	Optimised experimental conditions	Catalytic method	Active species	Oxidation efficiency	Recycling	Ref.
wb@FeO	MO	*T* = 40 °C, pH = 6, MO = 0.05 mM, catalyst = 10 mg	Photocatalytic degradation	e^−^	94.00%	10	155
Ag@S-nZVI/AC	NDMA	*T* = 24.85 °C, pH = 7, NDMA = 300 µg L^−1^, calalyst = 2.0 g L^−1^	Oxidative/Reductive	H^+^, O_2_^−^	—	4	129
CV-Fe^0^@Fe_2_O_3_	SPD	*T* = 25 °C, pH = 0–1, SPD = 5 mg L^−1^, catalyst = 10 mg	Oxidative degradation	SO_4_˙^−^, ˙OH	99.00%	—	156
EF/ZVI/PMS	TC-HCl	*T* = 80 °C	Oxidative degradation	HSO_5_^−^, SO_5_−	76.15%	5	157
nZVI/ALC	Cr^6+^, CIP	*T* = 80 °C, time = 6 h	Electrochemical degradation	˙OH, ˙O^2^, ^1^O_2_	99.90%, 89.90%	—	158
S-nZVI	NB	*T* = 700 °C, time = 2 h	Physio-chemical degradation	S^2−^, S_2_^2−^	—	—	159
GO/nZVI	MB	*T* = 30 °C, pH = 3 MB = 0.84%, catalyst = 200 mg L^−1^	Oxidative degradation	˙OH	99.99%	5	160
FA-ZVIbm	CdII-EDTA	pH = 7, CdII-EDTA = 50 ml, catalyst = 0.45g	Oxidative degradation	ROS	—	—	161
S_0.1_-nZVI@SS	TCEP	pH = 5.5, time = 10 h	Reductive degradation	S_2_^2−^	99.60%	—	162
OA-S-ZVI	LEV	H_2_O_2_ = 0.49 mM, PMS = 0.2 g L^−1^, catalyst = 0.75 g L^−1^	Oxidative degradation	SO_4_˙^−^, ˙OH	77.14%, 80.00%	10	163
nZVI@AC	DDBAC	pH = 7, DDBA = 10 mg L^−1^, catalyst = 0.5 g L^−1^	Oxidative degradation	SO_4_˙^−^, O_2_˙^−^	90.00%	4	164
ISBC	AA	pH = 3, AA = 5 mg L^−1^, catalyst = 0.1 g L^−1^	Oxidative degradation	SO_4_˙^−^, ˙OH	99.00%	—	165
SB-S-nZVI	TA	pH = 7, TA = 1000 mg L^−1^, catalyst = 0.4 g L^−1^	Oxidative degradation	˙O_2_^−^, ^1^O_2_, SO_4_˙^−^, ˙OH	99.31%	—	166
NBC-nZVI	NOR	*T* = 25 °C, pH = 5, NOR = 10 mg L^−1^, Catalyst = 0.15 g L^−1^	Oxidative degradation	˙O_2_^−^, SO_4_˙^−^, ˙OH	98.40%	3	167
S-ZVI/NaBrO_3_	SDZ	*T* = 25 °C, pH = 1, SDZ = 0.08 mM, catalyst = 0.07 g L^−1^	Oxidative degradation	Br_2_˙^−^, ROS	99.90%	5	168
S-nZVI@CD/PMS	NOR	NOR = 1.6 µM, catalyst = 0.05 g L^−1^	Oxidative degradation	SO_4_˙^−^, ˙OH	100%	11	169
S-nZVI/BC	4-CA	*T* = 25 °C, 4-CA = 50.0 µmol· L^−1^, catalyst = 0.55 g L^−1^	Oxidative degradation	SO_4_˙^−^, ˙OH, O_2_˙^−^	98.86%	3	170
S-nZVI/BC	PCA	*T* = 25 °C, PCA = 50 µmol L^−1^	Oxidative degradation	SO_4_˙^−^, ˙OH, O_2_˙^−^	96.43%	3	171
FeOXbm/Ni	BDE-47	*T* = 25 °C, BDE-47 = 1 mg L^−1^	Reductive degradation	H*ADS	76.71%	—	172
mZVI	PFOS	pH > 7, PFOS = 6 mg L^−1^, catalyst = 10 g L^−1^	Adsorption	F^−^	—	—	173

**Table 7:** Applications of nZVI adsorbents in water treatment

Adsorbents	Adsorbate	*Q*_max_ (mg g^−1^)	Adsorption isotherm	Mechanism	Kinetic model	Thermodynamic	Ref.
Ag@S-nZVI/AC	NDMA *N*-nitrosodimethylamine	0.0148	Freundlich	Physisorption	PSO	Endothermic	129
PANI-SA/ZVI	AsO_4_^3−^	104.167	Langmuir	Physisorption	PSO	Endothermic	174
nZVI-CS-Cu	RV5	52.91	Langmuir	Physisorption	PSO	Exothermic	175
ZVI/ILs	Cr^6+^	25.20	Freundlich	Chemisorption	PFO	—	176
nZVI	Cr^6+^	77.82	Langmuir	Chemisorption	PSO	—	177
ZIF-8@nZVI	Cr^6+^	57.70	Langmuir	Chemisorption	PSO	Endothermic	178
PU@nZVI	Cr^6+^	600.00	Freundlich	Physisorption	PSO	Exothermic	179
HTCTSIP-5	Cr^6+^	532.35	Langmuir	Electrochemical	PFO	—	180
nZVNi/nZVI-PDA@PVDF	Cr^6+^	75.65	Langmuir	Chemisorption, physisorption	PSO	Exothermic	181
ZVI@SBC	Cr^6+^	150.83	Langmuir	Chemisorption	PSO	—	182
P-NZVI	Cr^6+^	44.47	Langmuir	Chemisorption	PSO	Endothermic	15
SnZVI–BC–NH_2_	Cr^6+^	158.10	Langmuir	Physisorption	PSO	—	183
Fe^0^@N-PCM-H_2_O_2_	Cr^6+^	847.50	Freundlich	Chemisorption	PSO	—	184
S-nZVI/Ti_3_C_2_Tx	Cr^6+^	674.40	Langmuir	Chemisorption	PSO	—	185
EWF-nZVI	CR, RB	714.29, 68.49	Langmuir	Physisorption	PSO	Endothermic	186
BCP-nZVI	Co^2+^, Sr^2+^	107.10, 64.96	Langmuir	Chemisorption	PSO	—	187
BMJR-nZVI	SB	98.00	Langmuir	Physisorption	PSO	—	188
CnZVI	Ni^2+^	14.38	Freundlich	Physisorption	PFO	Exothermic	189
ACC-CH-NZVI	Phenol	29.94	Langmuir	Physisorption	PSO	Endothermic	190
rGOA-nZVI	MB, MO	3918, 667	Langmuir	Physisorption	PSO	Endothermic	191
NZVI-LBC	MB	1959.94	Freundlich	Physisorption	PSO	Endothermic	192
Fe-doped OMS-2	AB 62	54.00	Langmuir	Chemisorption	PSO	Exothermic	193
ZVI-DMSN	MB, DR 80	126.33, 69.57	Langmuir	Physisorption	PSO	Endothermic	194

The data summarized in the tables on catalytic (Table 6) and adsorption (Table 7) applications clearly highlight the versatility and performance of nZVI-based materials across diverse environmental remediation processes. Catalytic applications, particularly those involving advanced oxidation processes, consistently demonstrate rapid degradation of dyes, pharmaceuticals, and industrial pollutants, often achieving removal efficiencies exceeding 90% under optimized conditions. These systems commonly operate through Fenton-like pathways or ROS generation, where the high surface reactivity of nZVI especially when supported or surface-modified facilitates accelerated redox reactions. In contrast, the adsorption-focused studies presented in Table 7 emphasize the strong sorption capacities of nZVI composites for heavy metals and organic contaminants, with many materials exhibiting high Langmuir maximum capacities and fitting well to pseudo-second-order kinetics, indicating chemisorption–driven interactions. Collectively, both tables highlight a consistent trend: supported and modified nZVI systems outperform bare nZVI, owing to enhanced stability, reduced aggregation, improved dispersion, and synergistic interactions with the carrier materials. These findings reinforce the importance of material engineering such as employing biochar, clays, polymers, or hybrid nanostructures in optimizing nZVI performance and expanding its applicability in real-world water and wastewater treatment systems.

It is important to note that most reported removal efficiencies for green-synthesized nZVI are obtained under simplified laboratory conditions, typically using single-solute systems, acidic pH, and deionized water. In real wastewater matrices, competing ions (*e.g.*, bicarbonate, sulfate, natural organic matter) and fluctuating pH significantly reduce effective reactivity through surface fouling and rapid oxidation. Several studies report performance drops of 20–50% when transitioning from synthetic to real wastewater, underscoring the need for pilot-scale validation. Supported and sulfidated green nZVI systems show improved resistance to these matrix effects, but comprehensive long-term studies remain limited.

While much of the nZVI literature has focused on conventional pollutants such as heavy metals, chlorinated solvents, and synthetic dyes, there is growing recognition that nZVI-based materials must be evaluated against emerging contaminants of increasing regulatory and environmental concern. These include per- and polyfluoroalkyl substances (PFAS), endocrine-disrupting compounds (EDCs), pharmaceuticals and personal care products (PPCPs), and disinfection by-products such as nitrosamines.

PFAS, often termed “forever chemicals” due to their exceptionally strong C–F bonds and environmental persistence, represent a particularly challenging class of contaminants for nZVI-based remediation. Recent studies have demonstrated that nZVI can achieve significant PFOA sorption, with sorption capacities 2–4 orders of magnitude higher than those reported for soils and iron oxides, driven primarily by hydrophobic interactions between the perfluorocarbon chains and the nZVI surface.^195^ Sulfidated nZVI (S-nZVI) has shown enhanced resistance to surface passivation and improved PFAS adsorption performance even after prolonged aging. Moreover, immobilized S-nZVI@LDO composites have recently been employed for the simultaneous removal of PFOA and trichloroethylene (TCE) co-contaminants from groundwater, illustrating the potential of nZVI-based systems for treating mixed PFAS-chlorinated solvent matrices.^196^ However, the reductive defluorination of PFAS by nZVI remains thermodynamically and kinetically limited under ambient conditions, and most reported removal mechanisms are adsorption-driven rather than degradative, highlighting a critical knowledge gap.

Endocrine-disrupting compounds (EDCs), including bisphenol A (BPA), bisphenol S (BPS), 17β-estradiol (E2), and 17α-ethinylestradiol (EE2), pose significant risks to aquatic ecosystems and human health even at trace concentrations. nZVI-activated persulfate (nZVI/PS) systems have emerged as a promising advanced oxidation approach for EDC degradation. Roy *et al.* developed a green-synthesized Fc-rGO/nZVI nanocomposite using Punica granatum rind extract that achieved 94.4% BPA removal within 180 min.^197^ Sulfide-modified nZVI (S-nZVI) has demonstrated enhanced reductive removal of tetrabromobisphenol A (TBBPA), achieving over 90% degradation within 24 h—1.65 times higher than unmodified nZVI—and retaining 56% activity even after 11 week of aging.^198^ The S-nZVI/PS system has also been optimized for bisphenol S degradation under controlled conditions.^199^ These findings underscore the potential of modified nZVI platforms for addressing a broader spectrum of EDCs beyond conventional pollutants.

Similarly, nZVI has shown applicability for the removal of broader PPCPs and disinfection by-products. As discussed in Section 5, Riaz Ahmad *et al.* demonstrated that Ag@S-nZVI/AC composites could effectively remove the carcinogenic disinfection by-product *N*-nitrosodimethylamine (NDMA) through combined adsorption, redox, and hydrogenation pathways. Furthermore, cyclodextrin-supported sulfide zero-valent iron systems have been reported for the simultaneous removal of norfloxacin and antibiotic resistance genes (ARGs) from reclaimed water, representing a novel approach to addressing both chemical and biological emerging contaminants in water reuse scenarios.^169^ It is important to note that many studies on nZVI and emerging contaminants have been conducted under simplified laboratory conditions using single-solute, deionized water systems. In realistic wastewater and reclaimed water matrices, competing ions (*e.g.*, bicarbonate, sulfate, phosphate), natural organic matter, and pH fluctuations significantly reduce effective reactivity. Several studies report performance reductions of 20–50% when transitioning from synthetic to real wastewater, underscoring the urgent need for pilot-scale validation of nZVI-based systems against emerging contaminants in complex matrices.

## 8. nZVI performance *vs.* other nanomaterials and its environmental impacts

While nZVI is widely recognised for its effectiveness in environmental remediation, concerns have been raised regarding its potential adverse effects on ecosystems. Several studies have investigated the ecotoxicological and environmental implications of nZVI release. Toxicity studies have shown that elevated concentrations of nZVI can negatively affect various aquatic organisms. Notably, reduced survival rates, inhibited growth, and behavioural changes have been observed in fish, algae, and invertebrates exposed to nZVI.^200,201^ Such findings highlight the potential risks to aquatic ecosystems, especially under uncontrolled application scenarios.

In terrestrial environments, the impact of nZVI on soil microbial communities has also been documented. Research indicates that exposure to nZVI can alter microbial diversity, reduce microbial abundance, and affect enzymatic activities within the soil matrix.^202,203^ These shifts in microbial community structure can compromise soil health, potentially disrupting critical biogeochemical cycles and ecosystem services.

Further studies have examined the fate and transformation of nZVI in soil and groundwater. Over time, nZVI particles undergo oxidation, releasing Fe^2+^/Fe^3+^ ions and forming iron oxide nanoparticles.^204^ These transformations can influence both the mobility and long-term reactivity of the nanoparticles, thereby affecting the stability and efficiency of remediation processes.^205,206^ Collectively, these findings underline the importance of conducting comprehensive environmental risk assessments and developing responsible deployment strategies when using nZVI in field-scale applications.

Compared to other nanoscale zero-valent metals such as Cu, Ni, or Zn, nZVI offers an optimal compromise between high reductive reactivity and environmental safety. Its corrosion products mainly iron oxides and hydroxides are non-toxic and often beneficial for soil and water systems, whereas Cu and Ni nanoparticles may pose ecotoxicological risks even at low concentrations.

In addition, nZVI's surface chemistry is well understood, allowing rational modification through sulfidation, carbon or biochar encapsulation, and polymeric coatings to enhance selectivity and persistence. From a practical standpoint, the abundance and low cost of iron make nZVI economically and environmentally preferable for large-scale deployment.

A comparative evaluation of nZVI and other widely applied nanomaterials highlights the distinct advantages and limitations of each system in environmental remediation (Table 8). However, its tendency to oxidize, aggregate, and transform into less reactive iron oxides can reduce mobility and long-term performance. In contrast, iron oxide nanoparticles (Fe_3_O_4_/Fe_2_O_3_) offer greater chemical stability and easier magnetic recovery but display lower reductive capabilities.^207^ Carbon-based nanomaterials, including CNTs and graphene, provide high adsorption capacity/selectivity and tunable surface chemistry, yet their environmental persistence and potential toxicity raise regulatory concerns. Photocatalytic materials such as TiO_2_ are highly effective for degrading organic pollutants under light irradiation but have limited efficacy in dark subsurface environments. Alumina and silica nanoparticles typically act as inert adsorbents or support materials with relatively low ecotoxicity, whereas silver nanoparticles, despite their strong antimicrobial properties, exhibit high ecological toxicity due to Ag^+^ release. Overall, nZVI remains a cost-effective and powerful option for reductive remediation, but its environmental impacts and transformation behavior must be carefully managed relative to other nanomaterial alternatives.

**Table 8:** A comparison table of nZVI against several commonly used nanomaterials in environmental remediation and related applications

Attribute/material	nZVI	Iron oxides (Fe_3_O_4_/Fe_2_O_3_ NPs)	Carbon nanotubes (CNTs)/graphene	TiO_2_ nanoparticles	Nanoscale alumina/silica	Silver nanoparticles (Ag NPs)
Primary removal mechanisms	Reductive transformation, electron donation, adsorption	Adsorption, redox (less reductive), catalytic	Adsorption, π–π interactions, surface functionalization for catalysis	Photocatalytic oxidation, adsorption	Adsorption, support for catalysts	Antimicrobial action, adsorption, ion release
Reactivity (contaminant types)	Very high for reducible contaminants (halogenated organics, heavy metals—Cr(vi) reduction *via* Fe^0^)	Moderate; good for adsorption and fenton-like catalysis	High affinity for hydrophobic organics; can be functionalized for metals	High under UV/light for organics; limited in dark	Moderate; value as support rather than primary reactive phase	High for microorganisms; effective at low conc
Selectivity	Moderately selective — favors reducible species and strong electron acceptors	Less selective; broadly adsorbs polar and ionic species	Tunably selective *via* functional groups	Selective for organics under irradiation	Low intrinsic selectivity; depends on functionalization	Low chemical selectivity; biological targets primarily
Stability/persistence	Tends to oxidize/deteriorate to iron oxides; moderate persistence	Relatively stable (magnetic), persistent in environment	Highly persistent, very slow natural degradation	Very stable and persistent in sediments	Stable and persistent; often inert	Can dissolve/release Ag^+^; persistence depends on coating
Mobility in water/subsurface	Agglomerates—mobility limited unless stabilized (polymers, surfactants)	Moderate mobility if small and unaggregated	Low (agglomerates) unless functionalized	Low to moderate depending on size/coating	Low to moderate	Low to moderate; coating and salinity matter
Scalability/cost	Low–moderate cost; scalable synthesis; economical for large-scale treatment	Low cost; widely produced	High cost (especially high-quality CNTs/graphene)	Moderate cost; widely produced industrially	Low cost for common oxides	High cost relative to bulk materials
Regeneration/reuse	Difficult — Fe^0^ consumed; magnetic recovery possible but reactivity declines	Regeneration possible (chemical or thermal)	Possible if immobilized on support; regeneration varies	Regeneration *via* cleaning/thermal, photocatalytic activity sustained	Regenerable if used as support	Limited; antimicrobial effect may limit reuse
Byproducts/secondary impacts	Iron oxides/sulfides — may change redox and mobilize some elements; H_2_ can form under some conditions	None highly reactive; may catalyze fenton reactions producing ROS	Possible release of small carbon fragments; hydrophobic pollutants may persist	Reactive oxygen species (ROS) under light; potential toxicity to microbes	Generally inert; mechanical/colloidal effects possible	Release of Ag^+^ ions — toxic to microbes and aquatic life
Ecotoxicity concerns	Low-to-moderate; depends on dose, coating, and transformation products; can alter microbial communities	Low-to-moderate; can affect iron-cycling microbes and catalyze ROS	High concern for persistent, bio-persistent particulates and inhalation risk	Moderate — photocatalytic ROS can harm non-target organisms	Low (inert) but high loads can stress organisms *via* physical interactions	High — strong antimicrobial toxicity at low concentrations
Fate/transformation in environment	Rapid oxidation to iron oxides/hydroxides; may immobilize some contaminants or remobilize others through redox changes	Relatively stable; may adsorb contaminants long-term	Persistent; transport limited unless colloidal; may accumulate in sediments	Persistent; may aggregate/settle; activity depends on light	Persistent and largely inert; used as fixed media	Transforms by dissolution (Ag^+^), sulfidation reduces bioavailability
Regulatory/public perception	Mixed — seen as promising but regulators ask for fate/toxicity data	Generally accepted (iron oxides common in nature)	Increasing scrutiny due to persistence and inhalation risks	Regulated in some jurisdictions for nanoparticle discharge	Low regulatory attention but depends on application	High regulatory attention due to toxicity to microbes and aquatic life
Typical environmental applications	*In situ* groundwater remediation (permeable reactive barriers, injections), soil remediation	Adsorbents, magnetic separation, catalysts for fenton processes	Adsorption filters, membranes, electrode materials, sensing	Photocatalytic degradation of organics, self-cleaning surfaces	Support materials for catalysts, adsorbents, filtration media	Disinfection, antimicrobial coatings, limited remediation uses

## 9. Durability, reusability and aging behaviour of nZVI

The long-term performance of nZVI is strongly influenced by its tendency to oxidize, aggregate, and lose reactive surface area over time. Aging processes include the formation of Fe(ii)/Fe(iii) (hydr)oxide shells, structural transformation into magnetite or goethite, and particle agglomeration promoted by electrolytes or natural organic matter.^19,36,78,88,109,154^ These changes reduce the electron-transfer efficiency and limit the reusability of bare nZVI.

Several strategies have been proposed to improve durability. Regeneration methods such as mild acid washing, reductive reactivation with NaBH_4_ or Fe^2+^, and low-temperature heat treatments can partially restore surface reactivity, though they are rarely sustainable for large-scale applications. A more promising strategy is the use of surface modifiers and structural supports. For example, biochar, polymeric, or clay matrices physically stabilize nZVI and create microenvironments that inhibit oxidation while maintaining accessibility to contaminants.^78,112,132,154,208^ Similarly, sulfidation and carbon encapsulation have proven effective in slowing passivation and enhancing electron transfer during repeated use.^36,109^

The reusability of nZVI composites is system-dependent but generally remains high (60–90% efficiency retention after three to five cycles) when proper stabilization strategies are employed. This highlights that green synthesis routes often involving organic coatings derived from plant metabolites can inherently improve durability, as these biomolecules act as both reducing and capping agents that delay oxidation.

## 10. Future challenges and perspectives for nZVI-based technologies

Despite the substantial progress achieved in green synthesis and environmental deployment of nZVI, several critical challenges remain before this technology can transition from laboratory-scale demonstrations to reliable field implementation. Addressing these challenges requires coordinated advances in materials design, mechanistic understanding, environmental safety, and regulatory development.

(I) Scalability and reproducibility of green synthesis. Although plant- and biomass-mediated synthesis routes offer clear environmental advantages, their scalability is still constrained by variations in phytochemical composition, low reaction yields, and inconsistencies in particle quality. Future work should prioritize the development of standardized extraction protocols, metabolomic profiling to identify the most effective bioactive reductants, and process intensification strategies that enable continuous or semi-continuous production. Integrating green synthesis with support materials (*e.g.*, biochar, clays, natural polymers) can also improve particle dispersion and yield but requires optimization to maintain cost-effectiveness at industrial scales.

(II) Environmental impacts, safety, and long-term fate. The environmental behavior of nZVI including its transformation pathways, ecotoxicity, and long-term persistence remains insufficiently understood. While surface-modified, sulfonated, or polymer-coated nZVI has shown reduced toxicity compared to bare particles, systematic long-term studies in real soils, sediments, and complex wastewater matrices are still lacking. Developing biodegradable or environmentally adaptive coatings may further minimize ecological risks without compromising reactivity. Advanced tools such as high-resolution imaging, synchrotron spectroscopy, and reactive transport modeling are needed to elucidate nZVI ageing, mobility, and transformation under field-relevant conditions.

(III) Expanding the application scope through selective and hybrid systems. Conventional nZVI often displays non-selective reactivity, leading to competition with non-target species and reduced treatment efficiency in complex matrices. Future advancements should focus on engineering selective nZVI composites, including MOF–nZVI hybrids, polymer-encapsulated nZVI, and catalytic nZVI structures functionalized with specific ligands or dopants to enhance selectivity toward priority contaminants. Moreover, synergistic hybrid approaches combining nZVI with photocatalysis, biofiltration, microbial reductive systems, electrochemical oxidation, or adsorptive media represent a promising direction for maximizing treatment efficiency in heterogeneous waste streams.

In particular, expanding the application scope of green-synthesized nZVI to address recalcitrant emerging contaminants including PFAS, EDCs such as bisphenol analogues, and broader PPCPs should be considered a research priority. The development of nZVI-based materials capable of simultaneous removal of mixed contaminant systems (*e.g.*, co-occurring PFAS, heavy metals, and pharmaceuticals) under realistic wastewater conditions represents a critical frontier for next-generation remediation technologies. Integrating nZVI with advanced oxidation, membrane filtration, or biological processes may offer synergistic pathways for addressing these complex pollution scenarios.

(IV) Stability, mobility, and field performance. Rapid aggregation and surface passivation still limit the mobility and longevity of nZVI in subsurface systems. Future research should therefore explore robust stabilization frameworks, such as MOF-supported nZVI, polymer-based encapsulation, carbonaceous matrices, and hierarchical porous hosts. These architectures can maintain access to the reactive Fe^0^ core while mitigating oxidation and agglomeration, enabling sustained reactivity and improved field performance. Additionally, real-scale trials and pilot demonstrations are essential to validate laboratory observations under variable hydrogeochemical conditions.

(V) Regulatory and standardization gaps. The absence of unified guidelines governing the production, characterization, safe handling, and environmental release of engineered nanomaterials including nZVI represents a major barrier to commercialization. Collaborative efforts among researchers, industry stakeholders, and policymakers should aim to establish standardized testing protocols, environmental monitoring strategies, and risk benefit assessment frameworks. Such standardization is crucial for building public trust and ensuring responsible deployment.

(VI) Sustainability assessment and system-level integration. While green synthesis aligns with circular economy principles by utilizing renewable biomass, comprehensive LCA and TEA remain scarce. Future studies should incorporate full system-level evaluations from feedstock sourcing and energy use to waste generation and end-of-life behavior to ensure that green nZVI production is genuinely sustainable. Integrating agricultural waste valorization, renewable energy inputs, and closed-loop processing could further minimize the environmental footprint of nZVI technologies.

Overall, future advancements in nZVI research should move toward multifunctional, hybrid, and environmentally conscious systems. In particular, MOF–nZVI composites, polymer-engineered nZVI, and biochar-based architectures represent highly promising platforms for enhancing selectivity, stability, and catalytic performance. Combining these materials with biological and electrochemical processes may create next-generation remediation technologies capable of addressing complex environmental challenges. Ultimately, translating laboratory innovations into practical field solutions will require interdisciplinary collaboration, standardized methodologies, and a holistic consideration of environmental sustainability and societal acceptance. For example, future research should focus on standardized metabolomic profiling of plant extracts to quantitatively correlate specific phytochemical signatures with nZVI reactivity, stability, and aging behavior, coupled with pilot-scale validation of supported green nZVI systems in real wastewater matrices to assess long-term performance under realistic conditions.

## 11. Economic and environmental considerations in green synthesis

To date, quantitative economic and environmental assessments of green nZVI synthesis remain limited. Only a handful of studies have reported direct cost comparisons between plant-mediated and borohydride-based nZVI synthesis. Available data suggest that green routes can reduce reagent costs by utilizing low-cost plant biomass and eliminating the need for NaBH_4_ (approximately $50–120 kg^−1^ at laboratory scale), while operating under ambient conditions that lower energy input.^23,77,78^ However, no full-scale techno-economic analysis (TEA) has yet been published for green nZVI production, and the frequently cited “30–45% cost reduction” estimate is extrapolated from laboratory-scale reagent comparisons rather than from comprehensive process costing that includes biomass sourcing, extraction, quality control, and waste management. Similarly, life-cycle assessment (LCA) data comparing green and chemical nZVI remain scarce; to our knowledge, fewer than five peer-reviewed studies have attempted partial LCA of green nZVI, and none have provided a full cradle-to-grave analysis. The available partial assessments confirm reduced toxicity potential and lower hazardous waste generation for green routes, but have not quantified trade-offs related to land use, water consumption for biomass extraction, or end-of-life nanoparticle fate.

Green nZVI synthesis is frequently framed as aligned with circular economy principles and the UN Sustainable Development Goals (SDGs 6, 12, and 13). While this framing is conceptually reasonable—plant waste valorization reduces reagent costs and avoids hazardous by-products—it remains largely unvalidated by quantitative evidence. No study to date has demonstrated a closed-loop green nZVI production system at pilot or industrial scale, and claims of SDG alignment have not been supported by indicator-based sustainability assessments. Furthermore, the assumption that biomass-derived reducing agents are inherently “sustainable” overlooks potential burdens associated with seasonal supply variability, agricultural land competition, transportation logistics, and post-synthesis biomass waste disposal. These factors must be quantified through dedicated LCA and TEA studies before sustainability claims can be considered evidence-based rather than aspirational.

Three critical gaps currently prevent evidence-based sustainability evaluation of green nZVI: (i) the absence of standardized reporting metrics for extract composition, Fe precursor ratios, yield, and energy input, which hinders cross-study comparison; (ii) the lack of full cradle-to-grave LCA studies that account for biomass cultivation or collection, extraction, nZVI synthesis, application, and post-use fate; and (iii) the unavailability of pilot-scale TEA data that incorporate realistic process variables such as batch-to-batch variability, quality control costs, and scale-up losses. We recommend that future studies adopt a structured sustainability assessment framework comprising: (a) standardized mass and energy balances reported per gram of nZVI produced; (b) comparative LCA against borohydride-reduced nZVI using established impact categories (global warming potential, human toxicity, freshwater ecotoxicity); and (c) TEA including sensitivity analysis for key variables such as extract source, Fe salt cost, and production scale. Until such data become available, sustainability claims for green nZVI should be explicitly qualified as preliminary estimates rather than validated conclusions.

## 12. Conclusion

**Fig. 13:**
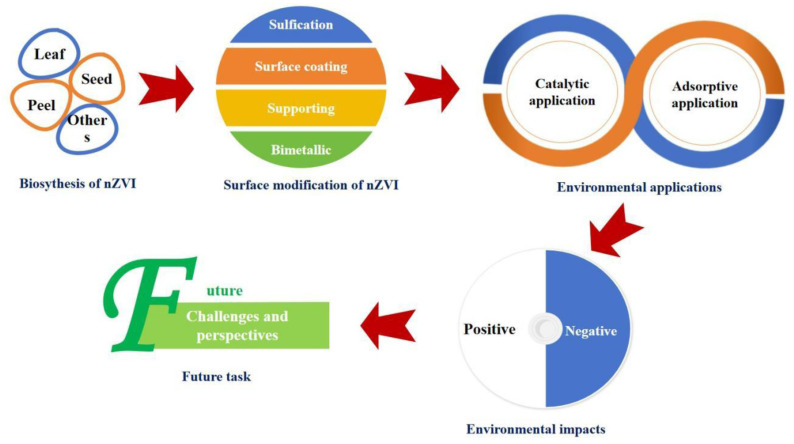
Conceptual framework of the review, illustrating the green synthesis routes of nZVI to environmental remediation.

This review summarizes recent progress in the green synthesis of nZVI and its applications in environmental remediation, emphasizing the interplay between Fe^0^/Fe^2+^/Fe^3+^ redox processes, sustainability, and circular-economy principles. Plant-extract-mediated synthesis remains the most promising route due to its low cost and tunable surface chemistry, while supported nZVI systems particularly those using biochar and polymers offer improved stability and reusability. Although notable advancements have been achieved, challenges persist regarding protocol standardization, reproducibility across biomass sources, and the scalability of green production. Furthermore, comprehensive LCA and TEA analyses are still needed to verify the environmental and economic feasibility of these methods. Future efforts should prioritize scalable, eco-safe synthesis, integration with microbial and waste-derived systems, and field-scale validation to advance green nZVI toward practical and sustainable deployment. The overall concept and key findings discussed in this review are visually summarized in the accompanying Fig. 13. The findings demonstrate that the true innovativeness of green-synthesized nZVI lies in its structure–function relationships and enhanced stability–reactivity balance, rather than in green synthesis alone.

## Author contributions

Basem E. Keshta: writing – original draft, supervision, resources, project administration, methodology, investigation, formal analysis, data curation. Dhiss Tesnim: writing – original draft. Jing Yu: writing – original draft, investigation. Qiaoping Kong: methodology, investigation, writing – review & editing. Huma Javeria: writing – review & editing. Yasmeen G. Abou El-Reash: writing – review & editing, funding acquisition. Heba G. El-Attar & Hany Koheil, Eida S. Al-Farraj & Mohamed N. Goda: investigation, writing – review & editing. Antonio Cid-Samamed: writing – review & editing.

## Conflicts of interest

The authors declare no conflict of interest.

## Data Availability

No Data has been used in this work.
